# Efficient Conversion of Light to Chemical Energy: Directional, Chiral Photoswitches with Very High Quantum Yields

**DOI:** 10.1002/anie.202005361

**Published:** 2020-06-08

**Authors:** Widukind Moormann, Tobias Tellkamp, Eduard Stadler, Fynn Röhricht, Christian Näther, Rakesh Puttreddy, Kari Rissanen, Georg Gescheidt, Rainer Herges

**Affiliations:** ^1^ Otto-Diels-Institut für Organische Chemie Christian-Albrechts-Universität zu Kiel Otto-Hahn-Platz 4 24118 Kiel Germany; ^2^ Institute of Physical and Theoretical Chemistry Graz University of Technology Stremayrgasse 9 8010 Graz Austria; ^3^ Institut für Anorganische Chemie Christian-Albrechts-Universität zu Kiel Max-Eyth-Str. 2 24118 Kiel Germany; ^4^ University of Jyvaskyla Department of Chemistry P.O. Box 35 40014 Jyväskylä Finland; ^5^ Smart Photonic Materials Faculty of Engineering and Natural Sciences Tampere University P. O. Box 541 33101 Tampere Finland

**Keywords:** diazocine, energy conversion, photochemistry, photochromism, quantum yields

## Abstract

Photochromic systems have been used to achieve a number of engineering functions such as light energy conversion, molecular motors, pumps, actuators, and sensors. Key to practical applications is a high efficiency in the conversion of light to chemical energy, a rigid structure for the transmission of force to the environment, and directed motion during isomerization. We present a novel type of photochromic system (diindane diazocines) that converts visible light with an efficiency of 18 % to chemical energy. Quantum yields are exceptionally high with >70 % for the *cis–trans* isomerization and 90 % for the back‐reaction and thus higher than the biochemical system rhodopsin (64 %). Two diastereomers (*meso* and racemate) were obtained in only two steps in high yields. Both isomers are directional switches with high conversion rates (76–99 %). No fatigue was observed after several thousands of switching cycles in both systems.

## Graphical abstract

**Azobenzene under lockdown**: Photoisomerizable molecules such as azobenzene convert light directly into chemical energy, however, with a low energy efficiency. By systematically reducing the conformational flexibility of azobenzene and thus preventing unproductive photochemical pathways, the energy conversion efficiency was increased from 1.4 % to 18.1 %, which is the largest of any known photochromic compound.

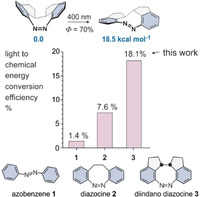

Photochemically induced *cis–trans* isomerizations (e.g. of retinal) are the key processes in the perception of light and for retinal‐based photosynthesis (pumping protons through ion channels in halophilic bacteria).^1^ Notwithstanding the very different functions (sensing and directed motion), both systems are based on the same reversible chemical reaction: the photoisomerization of retinal.^2^, ^3^, ^4^ Nature has optimized these systems for more than 2 billion years to achieve high quantum yields (64–67 %), conversion rates (between the (meta)stable states), and fatigue resistance.^5^ Within the last four decades a number of artificial photoswitches have been developed and optimized, aiming at a plethora of applications, such as motors, pumps, actuators, switchable drugs, sensors, and switchable liquid crystals. However, to the best of our knowledge, the quantum yields of biological systems are still unmatched (Figure 1)^6^ with the exception of diazocine, which was developed by our group.^7^

**Figure 1:**
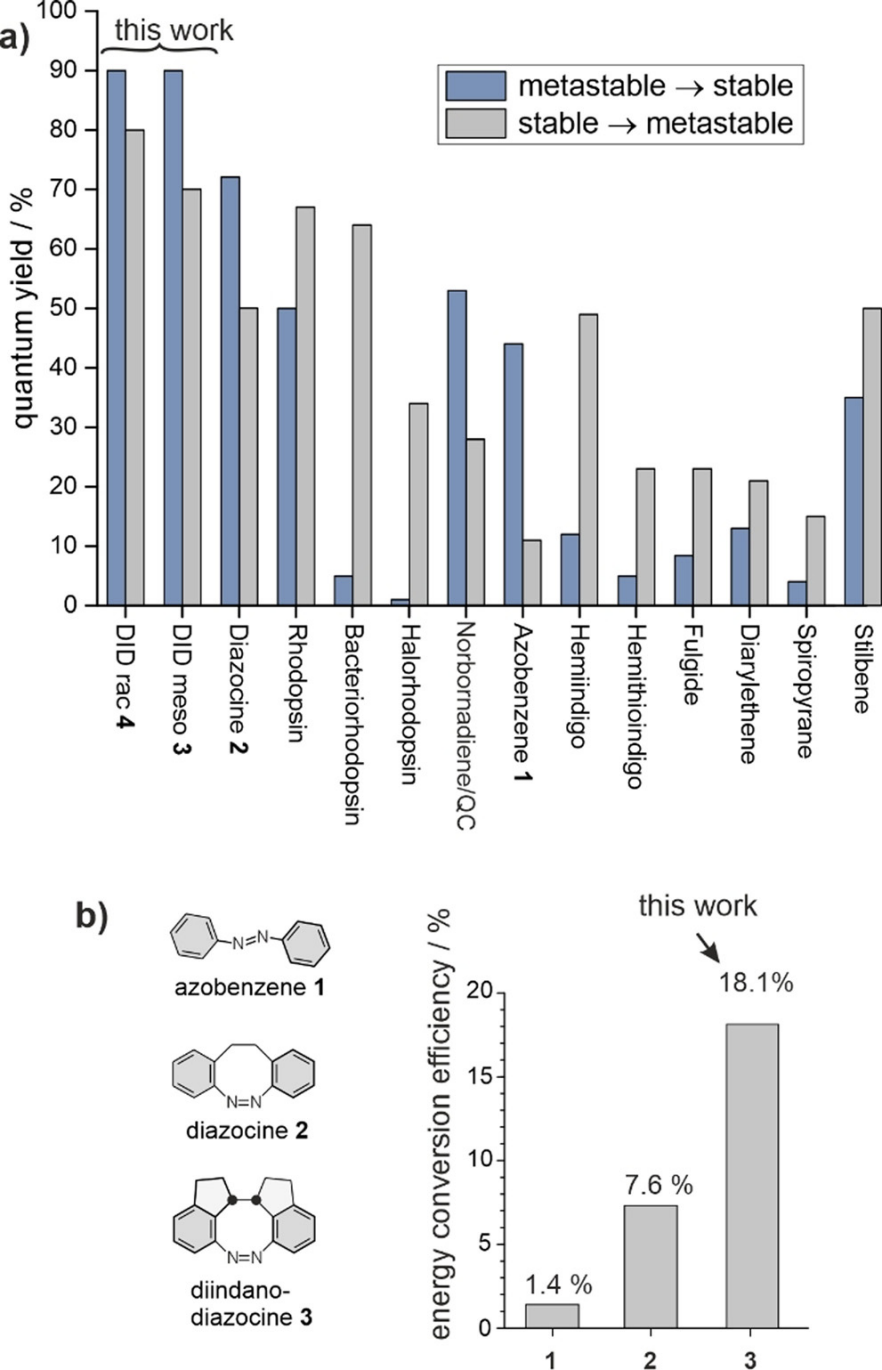
Switching properties of selected photochromic compounds. a) Quantum yields of diindane diazocines (DID *meso* **3** and DID *rac* **4**) presented in this work compared to frequently used artificial photoswitches and natural systems.^3^, ^4^, ^6^, ^8^, ^9^, ^10^, ^11^, ^12^, ^13^, ^14^ For an extended list of photoswitches and quantum yields see Supporting Information Table S1. b) Efficiency of the conversion of light to chemical energy (strain energy, see Supporting Information Figure S28).

Whereas previous artificial systems exhibit high conversion rates and fatigue resistance, their energy conversion process is not adequately efficient. The efficiency of converting light into chemical energy or to perform work is a function of the wavelength λ of the absorbed light, the quantum yield *Φ*, and the amount of energy *E* or work *W* produced. Particularly for the conversion of light into useful work, further properties such as directional motion (e.g. chiral switch) and sufficient rigidity for the transmission of the molecular force to the environment are prerequisites. Additional important parameters are conversion rates (photostationary states, PSS) and fatigue resistance (long‐term stability). We therefore set out to systematically improve the performance of the most frequently used artificial photoswitch azobenzene **1** and have been successful in enhancing all above parameters. The conversion wavelengths are shifted into the visible range, the quantum yields improved to 70–90 % (surpassing even the most efficient biological system rhodopsin), the strain energy produced increased to 18 kcal mol^−1^, the molecular switching movement is directional, the molecular framework is rigid (no conformational degrees of freedom), and the fatigue resistance amounts to at least a few thousand cycles.

Moreover, our new switches (**3** and **4**) presented here are accessible from commercially available starting materials in only two steps at high yields. Figure 1 b compares the efficiency of converting light into chemical energy (strain) of azobenzene **1** (1.4 %), diazocine **2** (7.6 %), and *meso*‐diindanediazocine **3** (18.1 %) upon irradiation at their peak absorption wavelengths 317, 385, and 400 nm. *meso*‐Diindanediazocine **3** is not suitable for immediate long‐term chemical energy storage;^15^ however, the short lifetime of the metastable state and the superior photophysical properties make it an ideal motor for ATP‐synthase type light‐driven synthesis^16^ and other light‐to‐chemical‐energy conversion systems.^17^

Arguably, the most frequently used artificial photoswitch is azobenzene **1**. Azobenzene derivatives are easily accessible, robust, reliable, chemically inert, and fatigue resistant. However, UV light is needed for the isomerization of the *trans* to the *cis* isomer, conversion rates are usually not high, and quantum yields are relatively low.^10^, ^18^ By the introduction of ethylene bridges between the *o,o′* positions of azobenzenes, we have been able to improve the photophysical properties considerably.^7^ Compared with **1**, these diazocines **2** exhibit enhanced quantum yields, switching wavelengths in the visible range, and very high conversion rates. Moreover, diazocines **2** are thermodynamically more stable in their bulkier *cis* configuration, which is of advantage in applications such as photopharmacology and mechanosensing.^19^, ^20^, ^21^, ^22^

However, neither the parent azobenzene **1** nor diazocine **2** are directional switches and are therefore unable to induce directional motion (Figure 2 a,b, curved arrows in **3** and **4** indicate the directionality of the molecular movement during isomerization). Introduction of additional bridges into the tricyclic diazocine system should further reduce conformational movements and introduces directionality into the switching motion. Moreover, the elimination of conformational degrees of freedom should concomitantly increase the quantum yields of the switching processes by preventing unproductive relaxation pathways.^23^, ^24^

**Figure 2:**
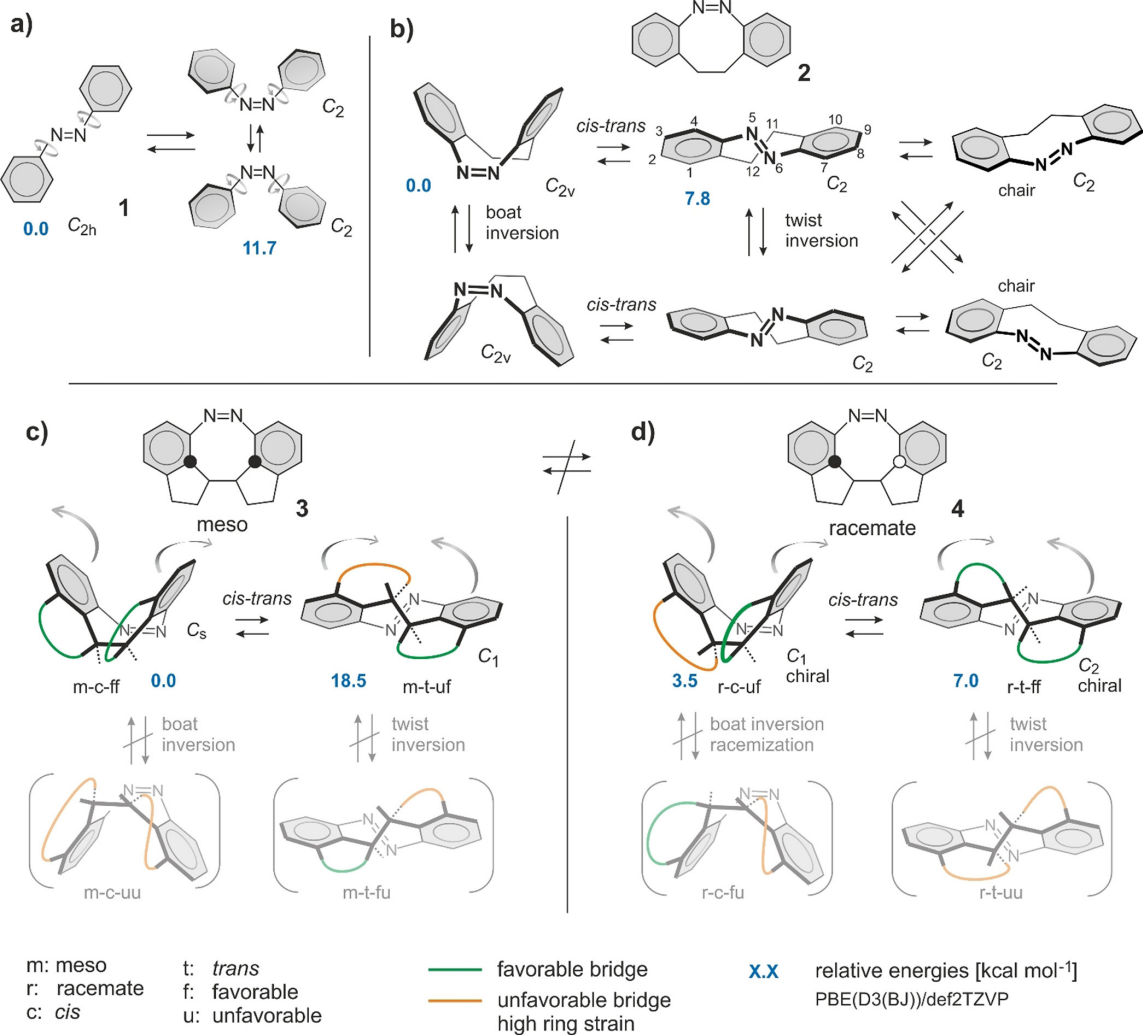
Stepwise reduction of conformational degrees of freedom by the introduction of bridges into azobenzene. a) azobenzene **1**, b) diazocine **2**, and c,d) doubly bridged diazocines (diindane diazocines) **3** and **4**. a) *trans* azobenzene **1** (*C*_2*h*_), upon irradiation, gives two enantiomeric *cis* azobenzenes **1** (*C*_2_) that rapidly interconvert at room temperature by almost free rotation of the phenyl groups. b) In diazocine **2** the phenyl rings are fixed by an ethylene bridge connecting the two rings. Conformational movements are restricted to twist inversions and twist‐chair inversions of the *trans* configuration. The *cis* configuration undergoes boat inversion at room temperature. c,d) There are two diastereomers of diindane diazocine: *meso* **3** and *racemate* **4**. Both compounds are conformationally rigid. There is no boat inversion and no twist inversion, which restricts each of the *cis* and the *trans* isomers to only one conformation. The −CH_2_−CH_2_− bridge is either favorable (*syn* annelation, green) or strained (*anti* annelation, orange). Relative energies (kcal mol^−1^) at the PBE(D3(BJ))/def2TZVP level of density functional theory are given in blue numbers. Structures m‐c‐uu and r‐t‐uu could not be located as minima on the potential energy surface, probably because of very high strain. The curved arrows in **3** and **4** indicate the directionality of the isomerization. Other molecular movements, for example, flapping in the reverse direction, are prohibited by the molecular framework.

Aiming at the improvement of our parent diazocine switches along the above lines, we pursued a design strategy based on ring strain. Figure 2 c illustrates our approach by means of a simplified model. Starting from the parent *cis* and *trans* diazocine **2** and introducing two ethylene bridges between positions 1–11 and 10–12, 8 different isomers of bridged diazocines (diindanodiazocines) are conceivable (Figure 2 c). Concomitant introduction of two stereocenters leads to a *meso* compound (m) **3** and a racemate (r) **4**. *Cis* and *trans* isomers are denoted with c and t. *Syn* annelations are favorable (f) and *anti* annelations are strained and unfavorable (u). Hence, four *meso* isomers result: m‐c‐uu, m‐c‐ff, m‐t‐uf, and m‐t‐fu. Analogously, the racemate comes as r‐c‐uu, r‐c‐ff, r‐t‐uf, and r‐t‐fu. *Meso* **3** and racemate **4** do not interconvert because that would imply an inversion at a sp^3^ carbon. Hence, the conformational degrees of freedom of meso **3** and *rac* **4** can be analyzed separately. Besides *cis*–*trans* isomerization, there two conceivable conformational transitions: ring inversion of the *cis*‐boat, and twist‐ and twist‐chair inversion of the *trans* isomer. Simple molecular model considerations reveal that the inversion at azo nitrogen atom 5 induces a rotation of the 12 methylene group and thus a change from a favorable (f) to an unfavorable bridge (u) or vice versa. Likewise the inversion at N6 (atom numbering see Figure 2 b) interconverts the pseudo axial and pseudo equatorial hydrogen atoms at position 11 and thus changes the strain of the corresponding bridge. Both boat inversion and twist inversion change both bridges from favorable to unfavorable or vice versa. From these simple model considerations, the reaction network shown in Figure 2 c,d can be derived. The relative energies of the 8 isomers can be estimated as well without explicit calculations. The *cis* isomer of diazocine is approximately 8 kcal mol^−1^ more stable than *trans*, and if we assume that an unfavorable bridge induces about 10 kcal mol^−1^ strain, the most stable *meso cis* structure m‐c‐ff is about 18 kcal mol^−1^ more stable than its *trans* isomer, whereas the *cis* (r‐c‐uf) and *trans* (r‐t‐ff) racemate should be almost isoenergetic. Structures m‐c‐uu and r‐t‐uu with two strained bridges are extremely unfavorable and probably not accessible. We should not observe the r‐c‐uf ⇄ r‐c‐fu ring inversion and the m‐t‐uf ⇄ m‐t‐fu twist inversion because a high activation barrier can be expected, since both conformational movements include the simultaneous change of the strain of two bridges. Explicit DFT calculations at the PBE(D3(BJ))/def2TZVP level of density functional theory (Supporting Information Figures S26 and S27, Table S7) support our qualitative picture (blue numbers in Figure 2 a–c). Upon irradiation, the *meso cis* compound (m‐c‐ff) should convert into a high‐energy *trans* isomer (m‐t‐uf) with a strain energy of 18.5 kcal mol^−1^. The *cis* and *trans* isomers of the racemate are close in energy (3.5 kcal mol^−1^ in favor of the *cis* isomer). Transition state calculations confirm the expected high barriers for boat and twist inversions (Figure 2 c,d, Supporting Information Figures S26 and S27). The bridging strategy in diindane diazocines **3** and **4** restricts the conformational degrees of freedom to only one conformation in each configuration.

The synthesis of the *meso* compound **3** and racemate **4** of diindane diazocine (DID) is quite straightforward. It includes only two steps starting from commercially available 4‐nitroindane **5**. Treatment of 4‐nitroindane **5** with *tert*‐butoxide and bromine afforded a mixture of the dinitro compounds **6** and **7**, which were separated by crystallization. The dinitro compounds **6** and **7** were converted into the diazocines **3** and **4** (Figure 3). The yields are 53 % for the *meso* compound **3** and 70 % for the racemic mixture **4**. An X‐ray crystal structure of the dinitro compound **7** reveals that the two nitro groups are suitably oriented for ring closure (Figure 3, top right), which explains the high yield (70 %) of the azo cyclization. The single‐crystal X‐ray diffraction study of diazocines **3** and **4** (Figure 3, bottom left and right, respectively, Supporting Information Table S6) proves the markedly different molecular geometries between the m‐c‐ff form of **3** and the r‐t‐ff form of **4**. The *cis* configuration of the ‐HC−CH‐ bridge with the H−C−C−H torsion angle of 39.3° in the diazocine **3** causes increased steric hindrance of the CH hydrogens and results in a slightly more twisted azo group (C−N=N−C torsion of 9.0°). The r‐t‐ff form of **4** is more relaxed with H−C−C−H torsion angle of 167.2° and C−N=N−C torsion of −4.6°.

**Figure 3:**
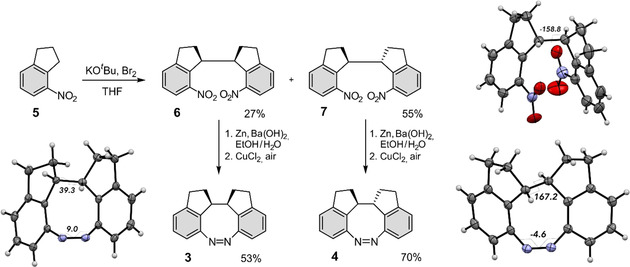
The diindane diazocines **3** and **4** were obtained by oxidative C−C coupling of 4‐nitroindane **5**, followed by reductive azo cyclization of the corresponding dinitro compounds **6** and **7**. The thermal ellipsoids of the X‐ray crystal structures are shown at the 50 % probability level.

The highly strained diindane diazocines **3** and **4** exhibit unusual properties regarding the half‐lives of their thermal relaxation. While the half‐life of the racemic mixture **4** is about four times longer than the half‐life of the parent system **2** (117 h vs. 27.6 h), the thermal relaxation for the *meso* compound **3** is about four orders of magnitudes faster than the parent system **2** (3 s). This is due to the high strain of the pincer‐shaped molecule **3**.

Half‐lives and photostationary states (PSS) were determined via NMR experiments in acetone (Table 1, Supporting Information Figures S13–S16). The half‐life of the *meso* compound **3** was extrapolated from low‐temperature measurements via Arrhenius plots (Supporting Information Figure S14). Additionally the thermal relaxation of the *trans*→*cis* isomerization was monitored via low‐temperature UV spectroscopy at 233 K (Figure 4 a). The UV spectrum of the *meso* diazocine **3** shows almost completely separated maxima for the n–π* transitions, (*cis* 411 nm, *trans* 468 nm), resulting in a PSS of 84 %, which is consistent with the PSS of the parent system **2** (Figure 4 b). The racemic diazocine **4** exhibits a *cis*→*trans* conversion of 76 % (385 nm). The n–π* transition for the *cis*→*trans* isomerization has a bathochromic shift resulting in a maximum at 431 nm and because the *trans*→*cis* n–π* transition is not shifted equally (478 nm), resulting in an overlap of absorption bands (Figure 4 b). *Trans*→*cis* conversions of **3** and **4** are achieved upon irradiation with green light (530 nm) and are quantitative within the limits of detection (UV and ^1^H NMR).

**Figure 4:**
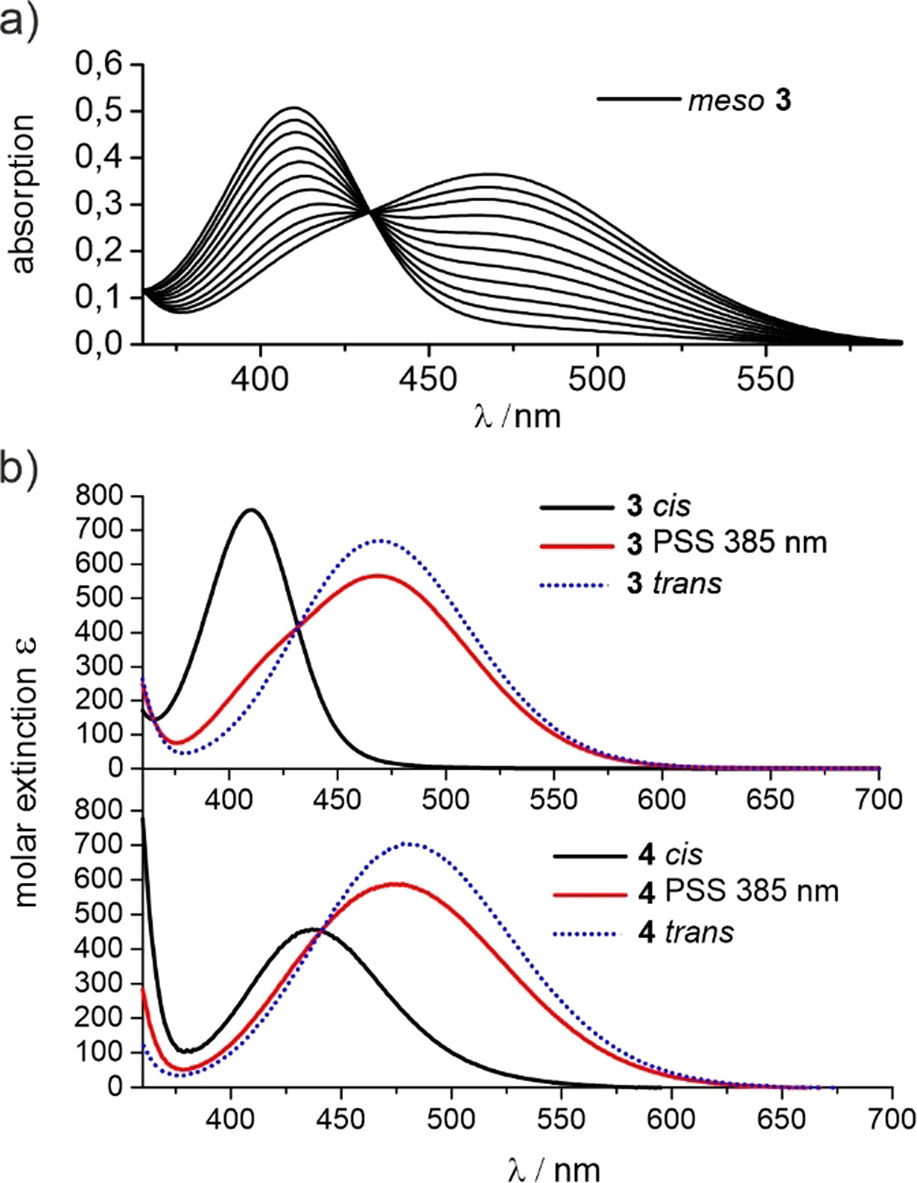
a) Selected UV spectra of the thermal relaxation of the meso diazocine **3** in THF at 233 K after irradiation at 385 nm. b) UV spectra of the n–π* transition of the diazocines **3** (top) and **4** (bottom) before and after irradiation at 385 nm in THF at 298 K. The UV spectra of the pure *trans* isomers **3** and **4** were obtained through extrapolation.

**Table 1:** Photophysical properties of DID **3** and **4** in comparison to the parent diazocine **2**.

Diazocine	*t*_1/2_ [h]	PSS (385 nm)	PSS (530 nm)	*Φ*_Z→E_ PSS (385 nm)	*Φ*_Z→E_ PSS (530 nm)
**2**	27.6^[a]^	87 %^[a]^	>99 %^[a]^	0.72^[b]^	0.9^[b]^
**3**	8.77×10^−4[a]*^	84 %^[c]^	>99 %^[c]^	0.7^[d]^	0.9^[e]^
**4**	117^[a]^	76 %^[e]^	>94 %^[e]^	0.8^[b]^	0.9^[b]^

[a] In acetone at 300 K. [b] In acetone at 293.15 K. [c] In acetone 233 K. [d] In acetone at 269.15 K, [e] In acetone at 298.15 K. * measured at five temperatures (233 K, 238 K, 240 K, 243 K) and extrapolated to room temperature.

The photostabilities of compounds **3** and **4** were determined via long‐term irradiation experiments. The photoswitching of the racemic diazocine **4** in acetonitrile was monitored via UV spectroscopy. The solution was irradiated at 385 nm and 530 nm for 30 seconds each in an alternating sequence. After 5000 cycles, UV spectra of several additional irradiation cycles were recorded showing no loss of absorption (Figure 5 a). The *meso* diazocine **3** was irradiated at 400 nm for 3.5 days (stirred solution 800 rpm, concentration: 1 mm, path length 1 cm, light intensity: 0.3 mW cm^−2^). This corresponds to ca. 2900 *cis*→*trans* isomerizations of one single molecule based on the molar absorption and the photon flux. The comparison of the UV spectra before and after illumination shows no sign of fatigue (Figure 5 b). The photochemical quantum yields of the isomerization of diindane diazocine **3** and **4** were measured in an online UV/Vis spectroscopy experiment (Supporting Information Figures S20–S25 and Tables S2–S4) as described by Rau and co‐workers.^25^, ^26^ Both diindane diazocines **3** and **4** have exceptionally high quantum yields confirming our strategy (Table 1). High‐temperature NMR measurements (Supporting Information Figures S17–S19) confirm the rigidity of the diindane diazocines **3** and **4** in comparison to the parent diazocine **2**. When diazocine **2** is heated from 298 K to 343 K the signal of the ethylene bridge protons is broadened, confirming a boat inversion at higher temperatures. The relevant signals of the diindane diazocines **3** and **4**, on the other hand, exhibit no broadening from 298 K to 343 K.

**Figure 5:**
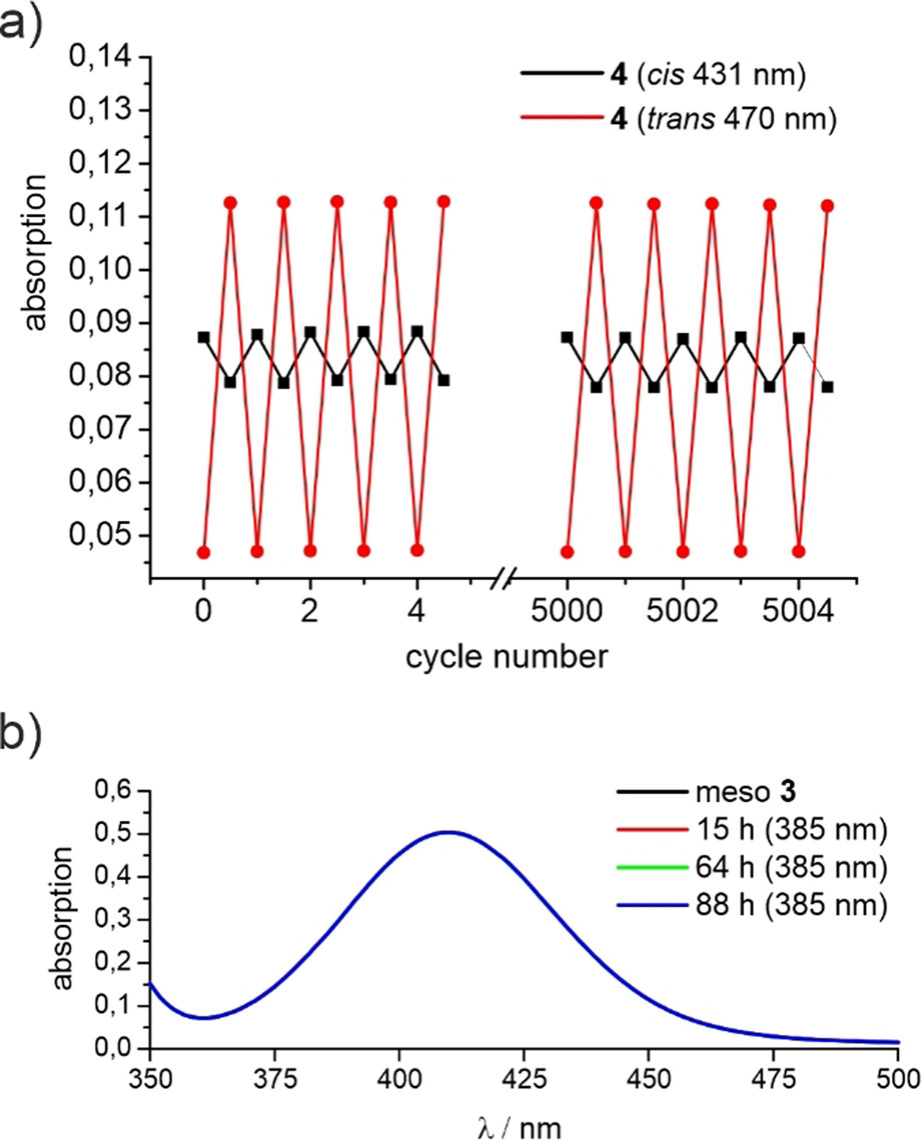
a) Absorption of the n–π* transitions of compound **4** at 431 nm (*cis*, black) and at 470 nm (*trans*, red) after alternating irradiation at 385 nm and 530 nm. b) UV spectrum of the *meso* compound **3** before (black) and after (blue) irradiation at 400 nm for 3.5 d.

Stepwise and systematic elimination of the conformational flexibility from azobenzene **1** to diazocine **2** drastically improved the photophysical properties. We introduced further bridges into the diazocine framework, preventing all conformational degrees of freedom leading to unproductive relaxation (boat, twist, and twist‐chair inversions) leaving only a well‐defined pincer‐type molecular movement for the *cis*→*trans* isomerization. The quantum yields of these diindanodiazocines **3** and **4** surpass even the natural systems rhodopsin, bacteriorhodopsin, and halorhodopsin. Concomitantly, we have been able to introduce considerable strain into the *meso* isomer of diindano diazocine **3**. The *trans* configuration is 18.5 kcal mol^−1^ higher in energy than the *cis* isomer. The high quantum yield (70 %) combined with the large ring strain that builds up upon *cis*→*trans* isomerization of *meso* diindanodiazocine **3**, leads to an exceptionally high light‐to‐chemical‐energy conversion efficiency of 18 %. The syntheses of *meso* **3** and *rac* **4** diindane diazocine include only two steps from commercially available chemicals. Multigram amounts can be prepared within a few days with standard laboratory equipment. Moreover, no fatigue has been observed over thousands of switching cycles. In contrast to azobenzene and most other photochromes, the molecular motion during isomerization is directional. Thus, *meso* diindanodiazocines (such as **3**) are ideal actuators to power molecular machines. *Rac* diindano diazocine **4** is the smallest chiral photoswitch known to date. It is also ideally suited as a component to switch chiroptical properties and to control the chirality of liquid crystals.^27^, ^28^, ^29^, ^30^

## Conflict of interest

The authors declare no conflict of interest.

## Supporting information

As a service to our authors and readers, this journal provides supporting information supplied by the authors. Such materials are peer reviewed and may be re‐organized for online delivery, but are not copy‐edited or typeset. Technical support issues arising from supporting information (other than missing files) should be addressed to the authors.

Supplementary

## References

[1] J. L. Spudich , Trends Microbiol. 2006, 14, 480–487. 17005405 10.1016/j.tim.2006.09.005

[2] V. I. Prokhorenko , A. M. Nagy , S. A. Waschuk , L. S. Brown , R. R. Birge , R. J. D. Miller , Science 2006, 313, 1257–1261. 16946063 10.1126/science.1130747

[3] T. Suzuki , R. H. Callender , Biophys. J. 1981, 34, 261. 7236851 10.1016/S0006-3495(81)84848-5 PMC1327470

[4] D. Oesterhelt , P. Hegemann , J. Tittor , EMBO J. 1985, 4, 2351. 15938054 10.1002/j.1460-2075.1985.tb03938.x PMC554509

[5] M. A. Ostrovsky , Paleontol. J. 2017, 51, 562.

[6] S. Wiedbrauk , H. Dube , Tetrahedron Lett. 2015, 56, 4266.

[7] R. Siewertsen , H. Neumann , B. Buchheim-Stehn , R. Herges , C. Näther , F. Renth , F. Temps , J. Am. Chem. Soc. 2009, 131, 15594. 19827776 10.1021/ja906547d

[8] A. Dreos , Z. Wang , B. E. Tebikachew , K. Moth-Poulsen , J. Andréasson , J. Phys. Chem. Lett. 2018, 9, 6174. 30296093 10.1021/acs.jpclett.8b02567 PMC6218103

[9] J. Tittor , D. Oesterhelt , FEBS Lett. 1990, 263, 269.

[10] H. M. D. Bandara , S. C. Burdette , Chem. Soc. Rev. 2012, 41, 1809. 22008710 10.1039/c1cs15179g

[11] C. Petermayer , H. Dube , J. Am. Chem. Soc. 2018, 140, 13558. 30303001 10.1021/jacs.8b07839

[12] K. Uchida , M. Irie , Chem. Lett. 1995, 24, 969.

[13] C. S. Santos , A. C. Miller , T. C. S. Pace , K. Morimitsu , C. Bohne , Langmuir 2014, 30, 11319. 25203491 10.1021/la503164e

[14] G. Tomasello , M. Garavelli , G. Orlandi , Phys. Chem. Chem. Phys. 2013, 15, 19763. 24141234 10.1039/c3cp52310a

[15] “Molecular systems for solar thermal energy storage and conversion”: K. Moth-Poulsen , Organic Synthesis and Molecular Engineering (Ed.: B. Nielson), Wiley, Hoboken, New Jersey, USA, 2014, Chapter 6, pp. 179–196.

[16] H. Sell , A. Gehl , D. Plaul , F. D. Sönnichsen , C. Schütt , F. Köhler , K. Steinborn , R. Herges , Commun. Chem. 2019, 2, 3468.

[17] X. Wang , F. Wang , Y. Sang , H. Liu , Adv. Energy Mater. 2017, 7, 1700473.

[18] E. Merino , Chem. Soc. Rev. 2011, 40, 3835. 21409258 10.1039/c0cs00183j

[19] G. Cabré , A. Garrido-Charles , À. González-Lafont , W. Moormann , D. Langbehn , D. Egea , J. M. Lluch , R. Herges , R. Alibés , F. Busqué , et al., Org. Lett. 2019, 21, 3780. 31070376 10.1021/acs.orglett.9b01222

[20] J. B. Trads , K. Hüll , B. S. Matsuura , L. Laprell , T. Fehrentz , N. Görldt , K. A. Kozek , C. D. Weaver , N. Klöcker , D. M. Barber , et al., Angew. Chem. Int. Ed. 2019, 58, 15421; 10.1002/anie.201905790 31441199

[21] P. Paoletti , G. C. R. Ellis-Davies , A. Mourot , Nat. Rev. Neurosci. 2019, 20, 514. 31289380 10.1038/s41583-019-0197-2 PMC6703956

[22] S. Li , G. Han , W. Zhang , Macromolecules 2018, 51, 4290.

[23] R. Siewertsen , J. B. Schönborn , B. Hartke , F. Renth , F. Temps , Phys. Chem. Chem. Phys. 2011, 13, 1054. 21072405 10.1039/c0cp01148g

[24] M. Böckmann , N. L. Doltsinis , D. Marx , Angew. Chem. Int. Ed. 2010, 49, 3382; 10.1002/anie.200907039 20340145

[25] E. Stadler , A. Eibel , D. Fast , H. Freißmuth , C. Holly , M. Wiech , N. Moszner , G. Gescheidt , Photochem. Photobiol. Sci. 2018, 17, 660. 29714365 10.1039/c7pp00401j

[26] H. Rau , G. Greiner , G. Gauglitz , H. Meier , J. Phys. Chem. 1990, 94, 6523.

[27] Y. Wang , Q. Li , Adv.Mater. 2012, 24, 1926. 22411073 10.1002/adma.201200241

[28] M. Mathews , N. Tamaoki , J. Am. Chem. Soc. 2008, 130, 11409. 18680250 10.1021/ja802472t

[29] R. A. van Delden , T. Mecca , C. Rosini , B. L. Feringa , Chem. Eur. J. 2004, 10, 61. 14695550 10.1002/chem.200305276

[30] M. J. Moran , M. Magrini , D. M. Walba , I. Aprahamian , J. Am. Chem. Soc. 2018, 140, 13623. 30293432 10.1021/jacs.8b09622

