# Supplementary material for: Efficient Conversion of Light to Chemical Energy: Directional, Chiral Photoswitches with Very High Quantum Yields
Source: Angew Chem Int Ed Engl. 2020 Jun 8;59(35):15081–6. doi: 10.1002/anie.202005361 (PMC7496762; doi:10.1002/anie.202005361)
Supplement: Supplementary file 1 — Supplementary [file ANIE-59-15081-s001.pdf]

Angewandte Chemie
Eine Zeitschrift der Gesellschaft Deutscher Chemiker

Supporting Information

Efficient Conversion of Light to Chemical Energy: Directional, Chiral Photoswitches with Very High Quantum Yields

*Widukind Moormann, Tobias Tellkamp, Eduard Stadler, Fynn Röhricht, Christian Näther, Rakesh Puttreddy, Kari Rissanen, Georg Gescheidt, and Rainer Herges**

anie_202005361_sm_miscellaneous_information.pdf

Table of Contents

1 **General Remarks** 1

2 **Quantum Yields of Frequently used Photoswitches** 2

3 **Syntheses** 6

4 **NMR Spectra** 12

5 **Photostationary states (PSS)** 16

6 **Thermal half-lifes** 19

7 **High Temperature NMR** 24

8 **Estimation of Isomerization Quantum Yields** 26

9 **Single Crystal Structure Determinations** 33

10 **Quantum Chemical Calculations** 35

11 **Summary of Photophysical Properties** 40

12 **Literatur:** 39

1 General Remarks

TLC was performed with TLC plates (Polygram Sil G/UV254), Co. Macherey–Nagel. Column chromatography was performed with silica gel 60 (0.04-0.063 mm) or aluminium oxide 60 basic (0.063-0.2 mm), Co. Merck. NMR spectra were recorded using a Bruker DRX 500 [1H NMR (500.1 MHz), 13C NMR (125.8 MHz)] without internal standard. Mass spectra were recorded on a MAT 8230 (EI, 70 eV), Co. Finnigan. IR spectra were recorded with a Perkin–Elmer 1600 series FT-IR spectrometer, using a golden-gate-diamond-ATR unit A531-G. UV/Vis spectra were recorded with a Lambda 14 UV/Vis spectrometer, Co. Perkin–Elmer. Elemental analyses were recorded with a CHNSO elemental analyzer EURO EA 3000 series, Co. Euro Vector. Irradiation was performed with LED light sources (385 nm: 12 x Nichia NCSU034A, FWHM = 9 nm, P(opt) = 12 x 340 mW, 530 nm: 16 x Luxeon LXML-PM01-0080, FWHM = 33 nm, P(opt) = 16 x 200 mW), Co. Sahlmann Photochemical Solutions.

2 Quantum Yields of Frequently used Photoswitches

Table S1: Quantum yields of frequently used photoswitches.

Compound	$\Theta_{E \rightarrow Z}$	$\Theta_{Z \rightarrow E}$	$\Theta_{ring\ closure}$	$\Theta_{ring\ open}$	Solvent	Ref.
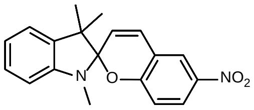			0.04	0.15	ethanol	1
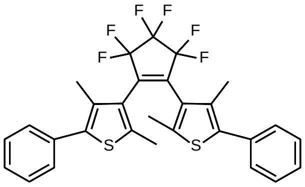			0.39	0.07	cyclo-hexane	1
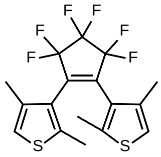			0.21	0.13	n-hexane	2
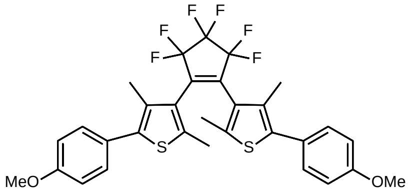			0.48	0.008	n-hexane	2
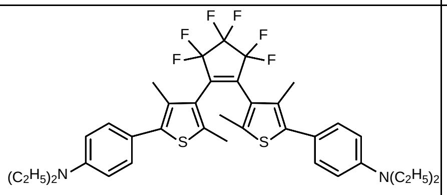			0.37	0.0025	n-hexane	2
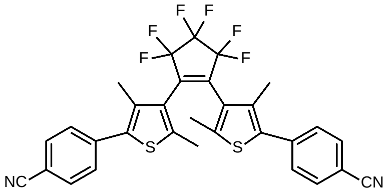			0.5	0.018	n-hexane	2
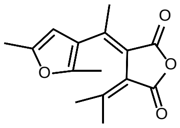		0.23	0.23	0.067	cumene	3
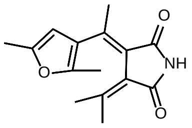		0.21	0.22	0.083	cumene	3
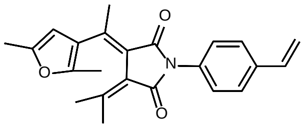		0.19	0.2	0.19	cumene	3

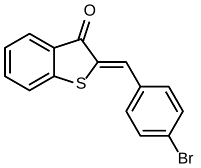	0.05	0.18			DCM	4
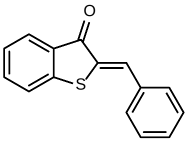	0.05	0.23			DCM	4
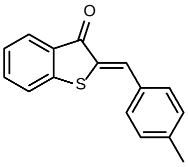	0.08	0.14			DCM	4
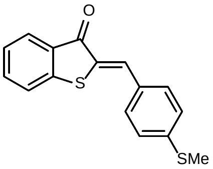	0.09	0.23			DCM	4
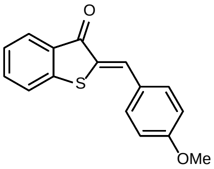	0.1	0.17			DCM	4
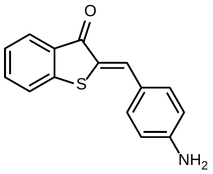	0.17	0.21			DCM	4
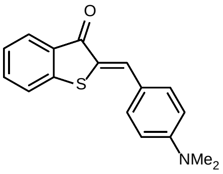	0.22	0.16			DCM	4
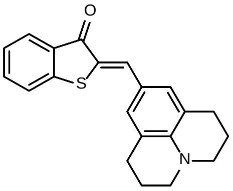	0.33	0.2			DCM	4
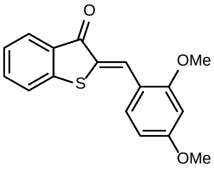	0.21	0.22			DCM	4
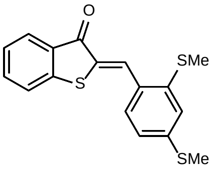	0.15	0.16			DCM	4

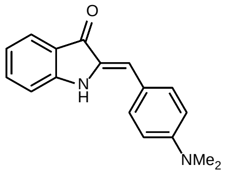	0.11	0.19			DMSO	5
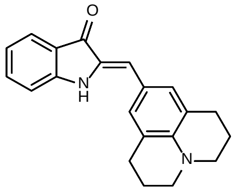	0.10	0.16			DMSO	5
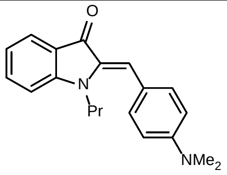	0.09	0.23			DMSO	5
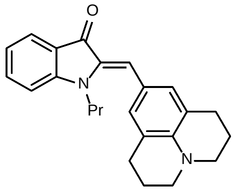	0.07	0.22			DMSO	5
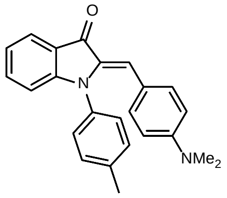	0.02	0.22			DMSO	5
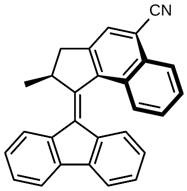	0.2				DCM	6
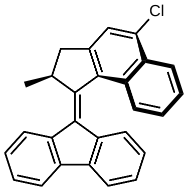	0.15				DCM	6
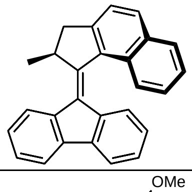	0.14				DCM	6
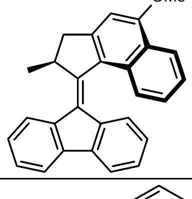	0.048				DCM	6
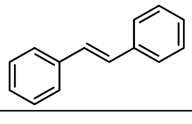	0.5[7]	0.35[8]	0.18[8]		methyl-cyclo-hexane,	7, 8

					cyclo-hexane	
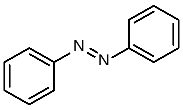	0.11	0.56			n-hexane	9
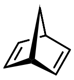			0.82			10
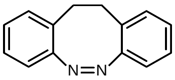	0.9	0.72			acetone	11
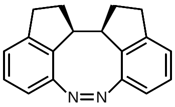	0.9	0.7			acetone	
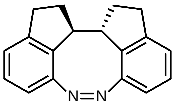	0.9	0.8			acetone	

3 Syntheses

(1*R*,1'*S*)-7,7'-dinitro-2,2',3,3'-tetrahydro-1*H*,1'*H*-1,1'-diindene and (1*R*,1'*R*/1*S*,1'*R*)-7,7'-dinitro-2,2',3,3'-tetrahydro-1*H*,1'*H*-1,1'-diindene

Under nitrogen atmosphere, 4-nitroindane (4.00 g, 24.5 mmol) was dissolved in dry THF (250 mL), cooled to 0 °C, followed by addition of potassium butoxide (4.12 g, 36.8 mmol). The reaction was stirred for 30 s before addition of bromine (1.5 mL, 29.4 mmol). After further stirring for 10 min, the reaction was added to 500 mL of ice/water. The precipitate was filtered and the filtrate extracted with CH_2Cl_2 (3 × 100 mL). The combined organic layers were washed with saturated sodium thiosulfate solution and saturated sodium chloride solution, then dried over MgSO4 and concentrated under reduced pressure. The crude product was purified by column chromatography (silica gel, *n*-pentane/DCM, 2:1) to afford a colorless solid.

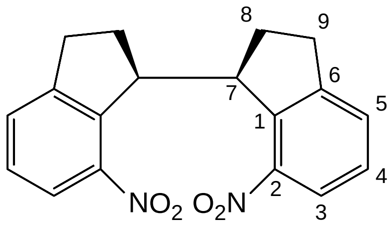

meso

yield: 740 mg (3.31 mmol, 27 %).

mp.: 176 °C.

R$_f$ = 0.11 (*n*-pentane/DCM, 2:1).

^{1}H-NMR (600.1 MHz, $CDCl_3$, 300 K): δ = 7.82 (d, ^3J = 8.0 Hz, 2 H, *H*-3), 7.38 (d, ^3J = 7.4 Hz, 2 H, *H*-5), 7.32 (ps. t, 2 H, *H*-4), 4.55 (d, ^3J = 8.9 Hz, 2 H, *H*-7), 2.74 (dd, ^2J = 16.5 Hz, ^3J = 9.8 Hz, 2 H, *H*-9$_a$), 2.47 (m$_c$, 2 H, *H*-9$_b$), 2.35 (m$_c$, 2 H, *H*-8$_a$), 2.02 (dd, ^2J = 13.4 Hz, ^3J = 8.1 Hz, 2 H, *H*-8$_b$) ppm.

^{13}C-NMR (150.9 MHz, $CDCl_3$, 300 K): δ = 148.56 (*C*-6), 147.23 (*C*-2), 140.00 (*C*-1), 130.39 (*C*-5), 128.46 (*C*-4), 122.85 (*C*-3), 49.26 (*C*-7), 32.13 (*C*-9), 30.01 (*C*-8) ppm.

MS (CI, isobutane): m/z (%) = 325 (100) $[M+H]^+$.

IR (ATR): $\tilde{\upsilon}$ = 3140 (w), 2943 (w), 2801 (w), 1519 (s), 1452 (m), 1348 (s), 1244 (m), 1222 (m), 1178 (m), 939 (w), 839 (m), 811 (m), 790 (m), 735 (s), 563 (m) cm^{-1}.

EA for $C_{18}H_{16}N_2O_4$ calcd.: C: 66.66, H: 4.97, N: 8.64 %;

found: C: 66.55, H: 4.99, N: 8.84 %.

(1*R*,1'*R*/1*S*,1'*R*)-7,7'-dinitro-2,2',3,3'-tetrahydro-1*H*,1'*H*-1,1'-diindene

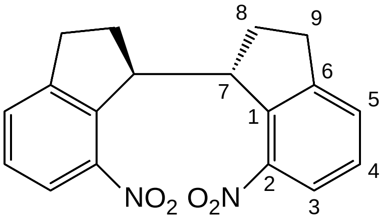

rac.

yield: 1.51 g (6.74 mmol, 55 %).

mp.: 181 °C.

R_f = 0.15 (*n*-pentane/DCM, 2:1).

^{1}H-NMR (600.1 MHz, $CDCl_3$, 300 K): δ = 7.66 (d, ^3J = 8.1 Hz, 2 H, *H*-3), 7.50 (d, ^3J = 7.4 Hz, 2 H, *H*-5), 7.30 (ps. t, 2 H, *H*-4), 4.28 (m_c, 2 H, *H*-7), 3.31 (m_c, 2 H, *H*-9_a), 2.39 (m_c, dd, ^2J = 16.1 Hz, ^3J = 8.5 Hz, 2 H, *H*-9_b), 2.32 (m_c, 2 H, *H*-8_a), 2.10 (dd, ^2J = 12.5 Hz, ^3J = 7.2 Hz, 2 H, *H*-8_b) ppm.

^{13}C-NMR (150.9 MHz, $CDCl_3$, 300 K): δ = 149.21 (*C*-6), 146.21 (*C*-2), 140.17 (*C*-1), 130.15 (*C*-5), 128.40 (*C*-4), 122.34 (*C*-3), 47.09 (*C*-7), 32.35 (*C*-8), 30.74 (*C*-9) ppm.

MS (CI, isobutane): m/z (%) = 325 (100) $[M+H]^+$.

IR (ATR): $\tilde{\upsilon}$ = 3145 (w), 2954 (w), 2808 (w), 1514 (s), 1447 (m), 1348 (s), 1243 (m), 1192 (w), 1130 (w), 938 (w), 864 (w), 812 (m), 790 (m), 742 (s), 557 (m) cm^{-1}.

EA for $C_{18}H_{16}N_2O_4$ calcd.: C: 66.66, H: 4.97, N: 8.64 %;

found: C: 66.79, H: 4.98, N: 8.77 %.

(12a*R*,12b*S*,*Z*)-1,2,11,12,12a,12b-hexahydrodiindeno[7,1-cd:1',7'-fg][1,2]diazocine

To a solution of 1*R*,1'*S*)-7,7'-dinitro-2,2',3,3'-tetrahydro-1H,1'H-1,1'-diindene (203 mg, 626 µmol) in EtOH (35 mL) were added an aqueous solution of barium hydroxide [$Ba(OH)_2\cdot 8H_2O$] (590 mg, 1.87 mmol) in H_2O (16 mL) and zinc powder (652 mg, 10 mmol), and the mixture was stirred for 6 h under reflux. The reaction mixture was filtered through Celite, and the solvent was removed under reduced pressure. The crude product was dissolved in CH_2Cl_2 and filtered through Celite, and the solvent was removed under reduced pressure. The crude product was dissolved in 0.1 M methanolic NaOH solution (30 mL), $CuCl_2$ (4 mg, 29.8 µmol) was added, and air was bubbled through the solution until completion of the reaction. The reaction was neutralized with 6 M HCl solution. After addition of saturated sodium bicarbonate solution, the aqueous layer was extracted with CH_2Cl_2. The combined organic layers were dried over $MgSO_4$ and the solvent was removed under reduced pressure. The crude product was purified by column chromatography (silica gel, *n*-pentane/DCM, 2:1) to afford a yellow solid.

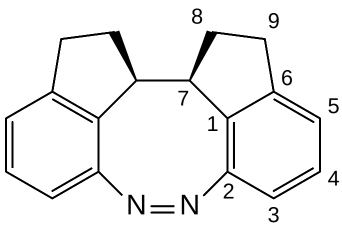

meso

yield: 114 mg (438 µmol, 70 %).

mp.: 171 °C.

$\mathbf{R_f}$ = 0.14 (*n*-pentane/DCM, 2:1).

^{1}**H-NMR** (500.1 MHz, $CDCl_3$, 300 K): δ = 7.14 (ps. t, 2 H, *H*-4), 7.01 (d, ^{3}J = 7.4 Hz, 2 H, *H*-5), 6.88 (d, ^{3}J = 7.8 Hz, 2 H, *H*-3), 3.58 (m_c, 2 H, *H*-7), 2.95-2.74 (m, 4 H, *H*-9), 2.26 (m_c, 2 H, *H*-8_a), 1.85 (m_c, 2 H, *H*-8_b) ppm.

^{13}**C-NMR** (125.8 MHz, $CDCl_3$, 300 K): δ = 152.27 (*C*-2), 145.19 (*C*-6), 133.22 (*C*-1), 127.39 (*C*-4), 123.42 (*C*-5), 118.35 (*C*-3), 47.44 (*C*-7), 32.68 (*C*-9), 29.81 (*C*-8) ppm.

MS (EI, 70 eV): m/z (%) = 260 (100) $[M]^+$, 231 (34), 217 (52), 202 (42), 115 (26).

MS (CI, isobutane): m/z (%) = 261 (100) $[M+H]^+$.

IR (ATR): $\tilde{\upsilon}$ = 2974 (w), 2934 (w), 2901 (w), 2841 (w), 1581 (w), 1523 (w), 1458 (m), 1427 (w), 1258 (w), 1157 (w), 1063 (w), 851 (w), 821 (w), 783 (m), 763 (s), 718 (m), 553 (w) cm^{-1}.

UV (MeCN): λ_{max} (lg ε) = 403 (2.86), 300 (3.39), 249 (3.73) nm.

EA for $C_{18}H_{16}N_2$ calcd.: C: 83.04, H: 6.19, N: 10.76 %;

found: C: 83.59, H: 6.26, N: 10.72 %.

(12a*R*,12b*S*/12a*S*,12b*R*,*Z*)-1,2,11,12,12a,12b-hexahydrodiindeno[7,1-cd:1',7'-fg][1,2]-diazocine

To a solution of 1*R*,1'*S*)-7,7'-dinitro-2,2',3,3'-tetrahydro-1H,1'H-1,1'-diindene (298 mg, 919 µmol) in EtOH (51 mL) were added an aqueous solution of barium hydroxide [$Ba(OH)_2 \cdot 8H_2O$] (868 mg, 2.75 mmol) in H_2O (24 mL) and zinc powder (958 mg, 14.7 mmol), and the mixture was stirred for 6 h under reflux. The reaction mixture was filtered through Celite, and the solvent was removed under reduced pressure. The crude product was dissolved in CH_2Cl_2 and filtered through Celite, and the solvent was removed under reduced pressure. The crude product was dissolved in 0.1 M methanolic NaOH solution (44 mL), $CuCl_2$ (6 mg, 43.8 µmol) was added, and air was bubbled through the solution until completion of the reaction. The reaction was neutralized with 6 M HCl solution. After addition of saturated sodium bicarbonate solution, the aqueous layer was extracted with CH_2Cl_2. The combined organic layers were dried over $MgSO_4$ and the solvent was removed under reduced pressure. The crude product was purified by column chromatography (silica gel, *n*-pentane/DCM, 2:1) to afford a yellow solid.

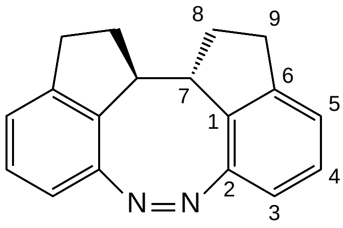

rac.

yield: 167 mg (643 µmol, 70 %).

mp.: 172 °C.

R_f = 0.17 (*n*-pentane/DCM, 1:1).

^{1}H-NMR (500.1 MHz, $CDCl_3$, 300 K): δ = 7.43 (d, ^3J = 8.0 Hz, 2 H, *H*-3), 7.29 (ps. t, 2 H, *H*-4), 7.15 (d, ^3J = 7.3 Hz, 2 H, *H*-5), 3.02-2.90 (m, 6 H, *H*-7, *H*-9a, *H*-9b), 2.21 (m_c, 2 H, *H*-8_a), 1.97 (m_c, 2 H, *H*-8_b) ppm.

^{13}C-NMR (125.8 MHz, $CDCl_3$, 300 K): δ = 148.95 (*C*-2), 144.71 (*C*-6), 133.11 (*C*-1), 127.22 (*C*-4), 125.97 (*C*-3), 124.74 (*C*-5), 49.56 (*C*-7), 31.69 (*C*-8), 31.66 (*C*-9) ppm.

MS (ESI-TOF, $CHCl_3$/MeOH): m/z (%) = 261 (100) $[M+H]^+$.

IR (ATR): $\tilde{\upsilon}$ = 2967 (w), 2910 (w), 2881 (w), 2323 (w), 1528 (w), 1495 (w), 1307 (w), 1158 (w), 869 (w), 818 (w), 785 (s), 756 (s), 691 (w), 666 (w) cm^{-1}.

UV (MeCN): λ_{max} (lg ε) = 434 (2.69), 314 (3.70), 257 (3.81) nm.

EA for $C_{18}H_{16}N_2$ calcd.: C: 83.04, H: 6.19, N: 10.76 %;

found: C: 82.40, H: 6.17, N: 10.59 %.

4 NMR spectra

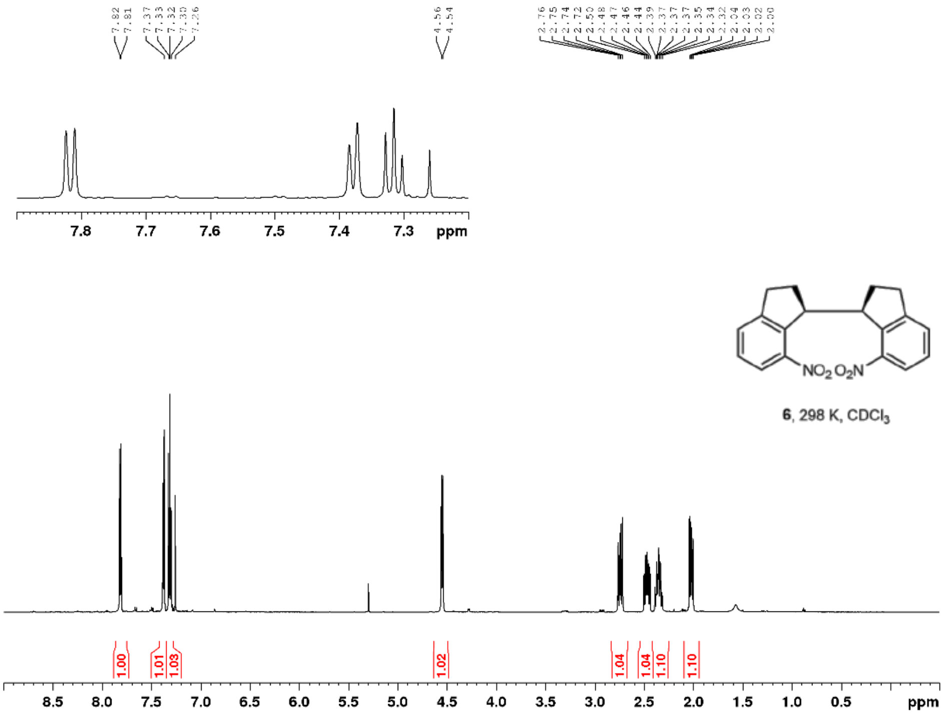

Figure S1: ^{1}H-NMR spectrum of compound **6** measured in deuterated chloroform.

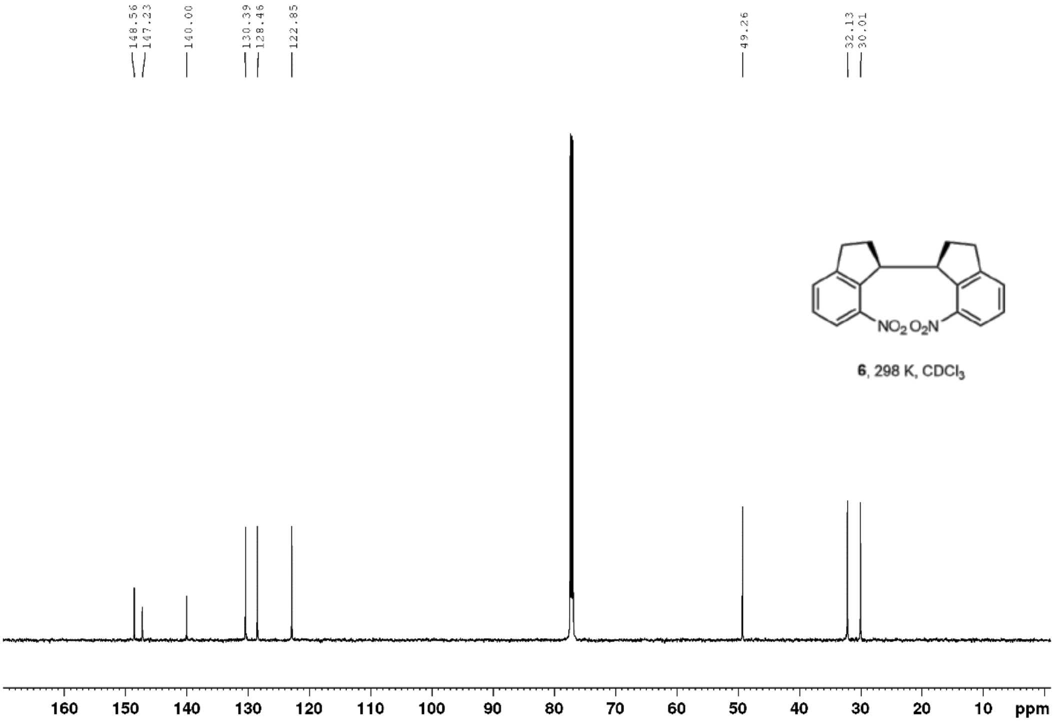

Figure S2: ^{13}C-NMR spectrum of compound **6** measured in deuterated chloroform.

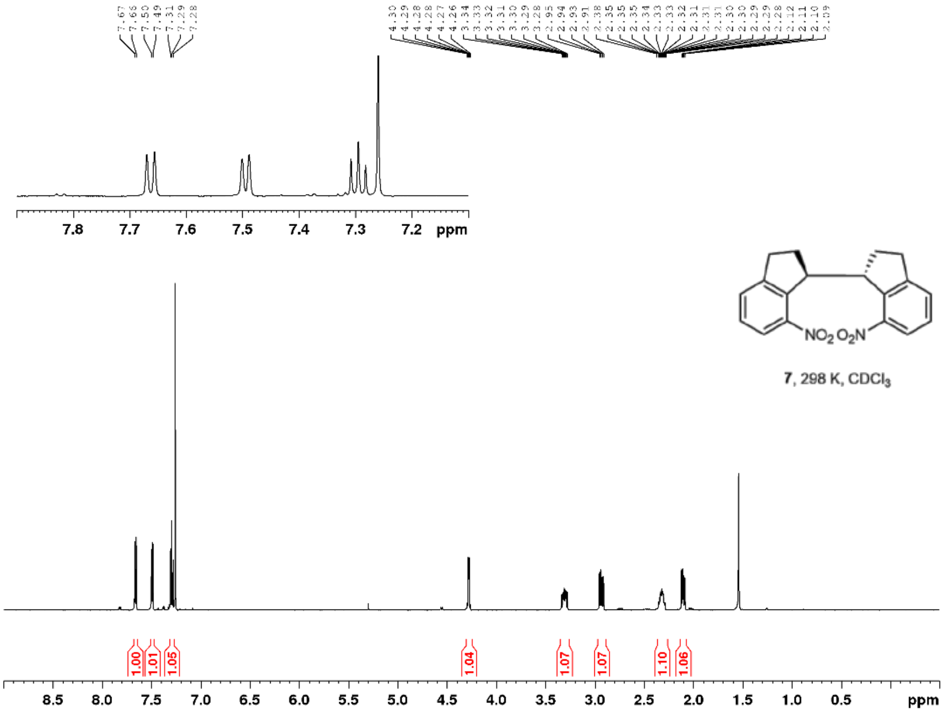

Figure S3: ^{1}H-NMR spectrum of compound **7** measured in deuterated chloroform.

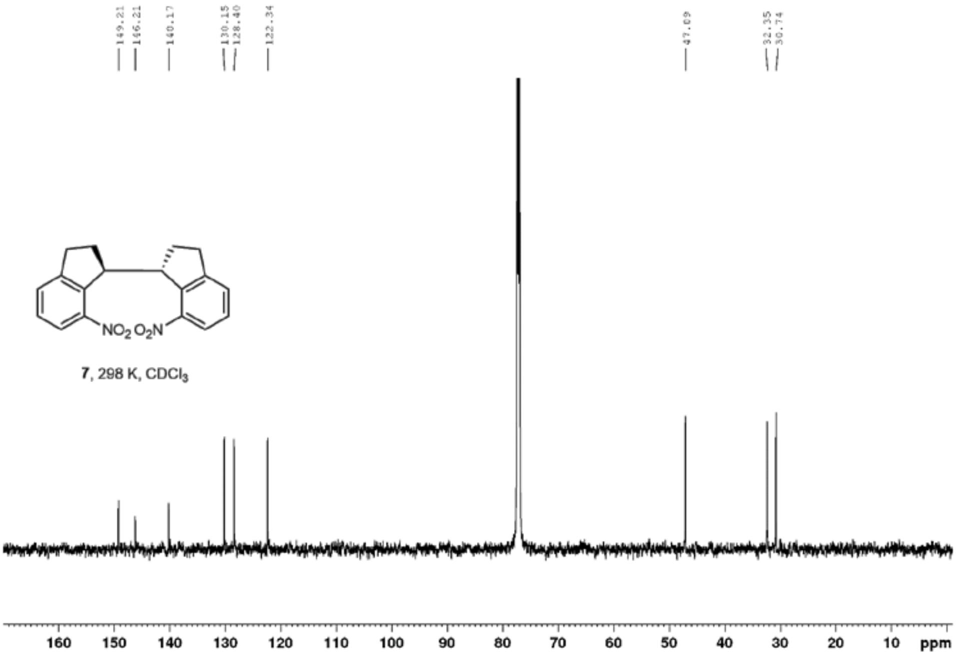

Figure S4: ^{13}C-NMR spectrum of compound **7** measured in deuterated chloroform.

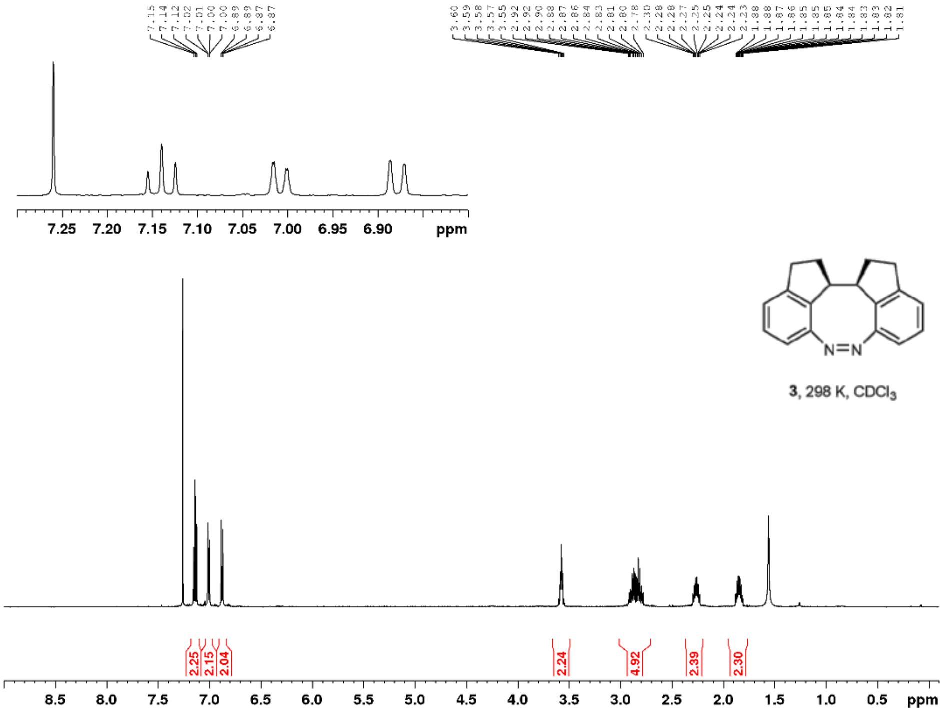

Figure S5: ^{1}H-NMR spectrum of compound DID meso **3** measured in deuterated chloroform.

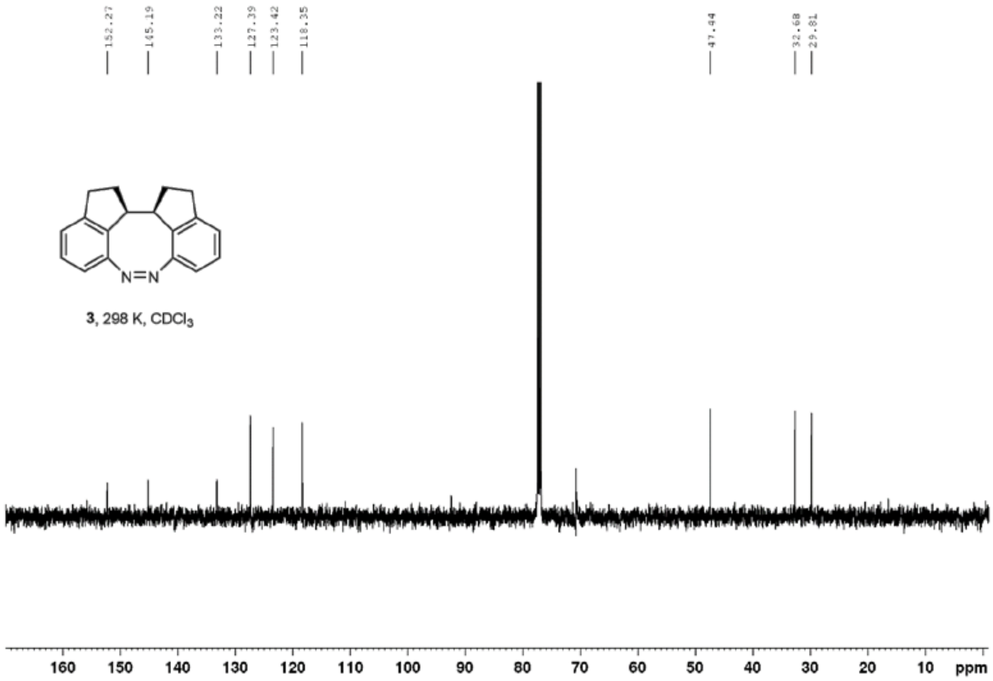

Figure S6: ^{13}C-NMR spectrum of compound DID meso **3** measured in deuterated chloroform.

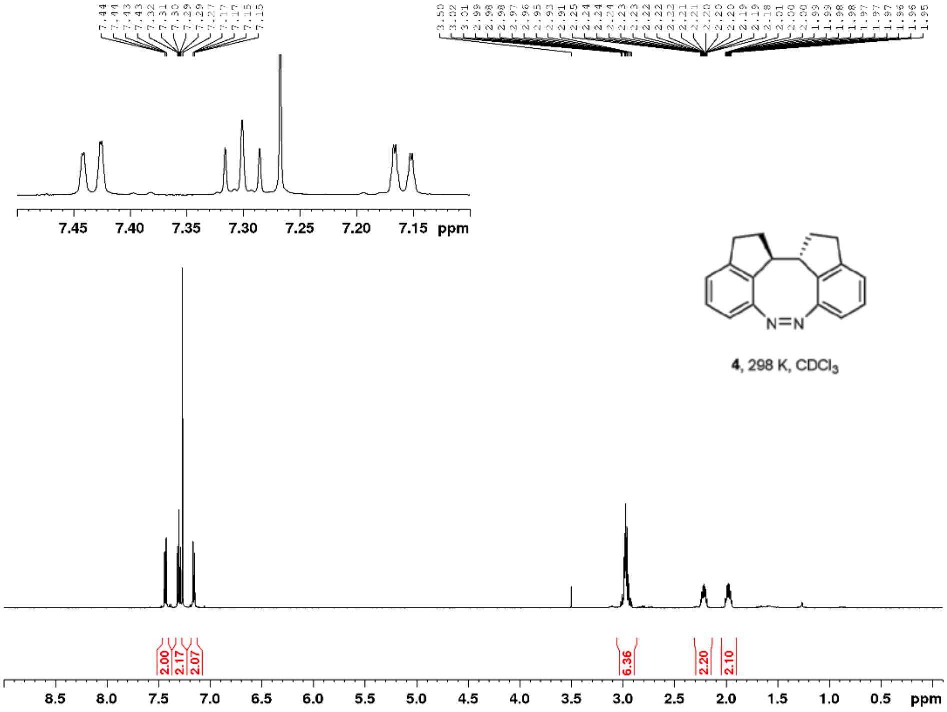

Figure S7: ^{1}H-NMR spectrum of compound DID rac **4** measured in deuterated chloroform.

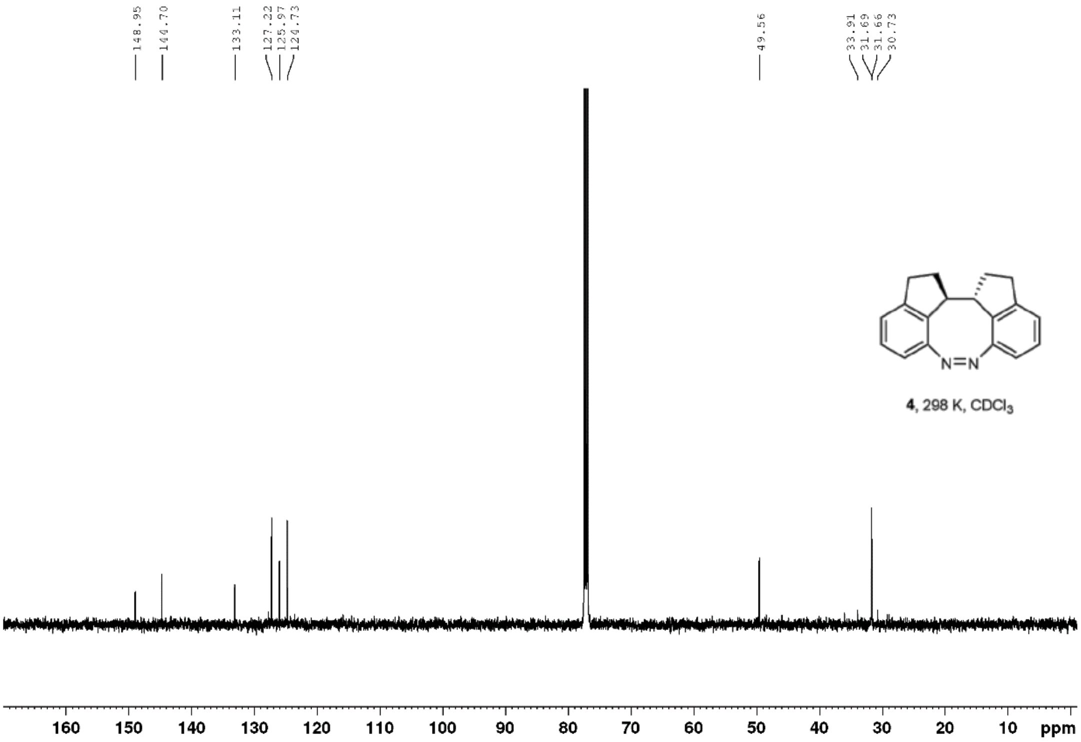

Figure S8: ^{13}C-NMR spectrum of compound DID rac **4** measured in deuterated chloroform.

5 Photostationary states (PSS)

Photostationary states (PSS) were investigated by ^{1}H-NMR spectroscopy and measured in acetone-d_6 at 233 K (DID meso) and 298 K (DID rac). Samples of 10 mM were prepared under exclusion of light and measured without irradiation. Afterwards the samples were irradiated for 5 min at 385 nm outside the NMR spectrometer. After recording the samples were irradiated again under the same conditions to guarantee no further isomerization and recorded a second time. Integration of the relevant signals in the ^{1}H-NMR spectra leads to the PSS at 385 nm.

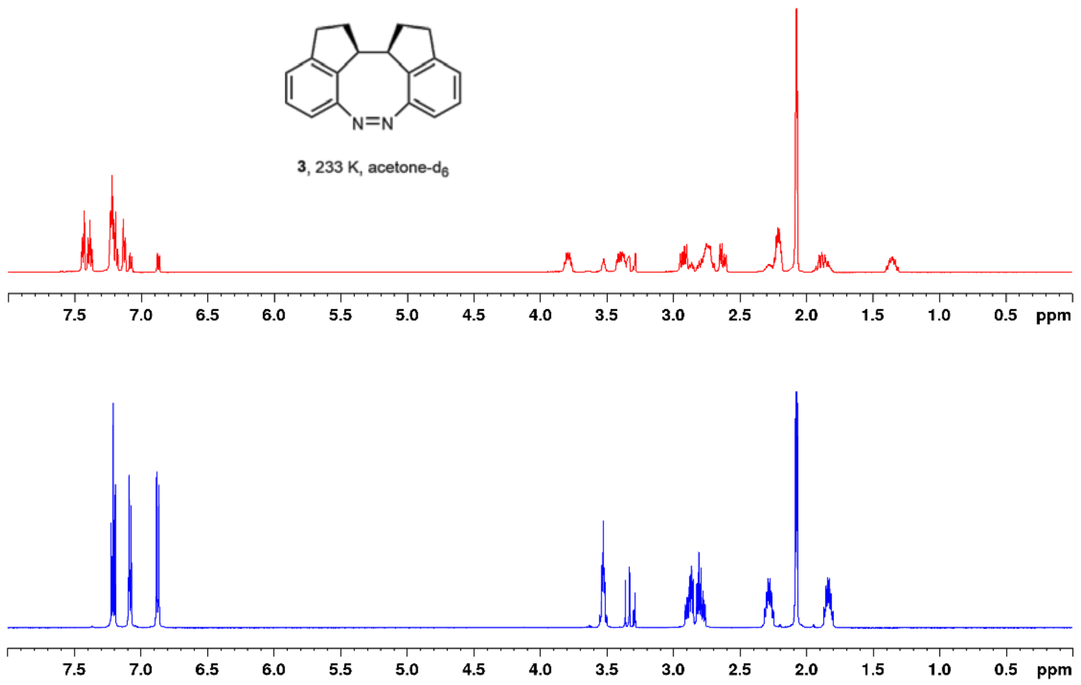

Figure S9: ^{1}H-NMR spectrum of compound DID meso **3** at PSS (385 nm) in red and PSS (530 nm) in blue both spectra measured in acetone at 233 K.

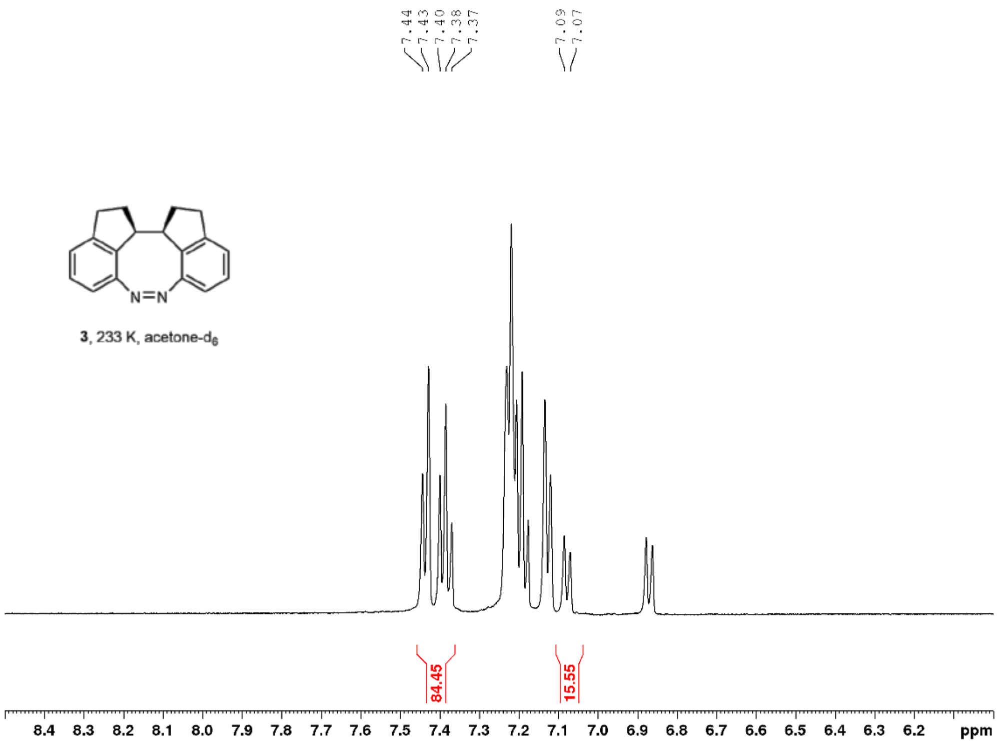

Figure S10: ^{1}H-NMR spectrum of compound DID meso **3** at PSS (385 nm) at 233 K in acetone.

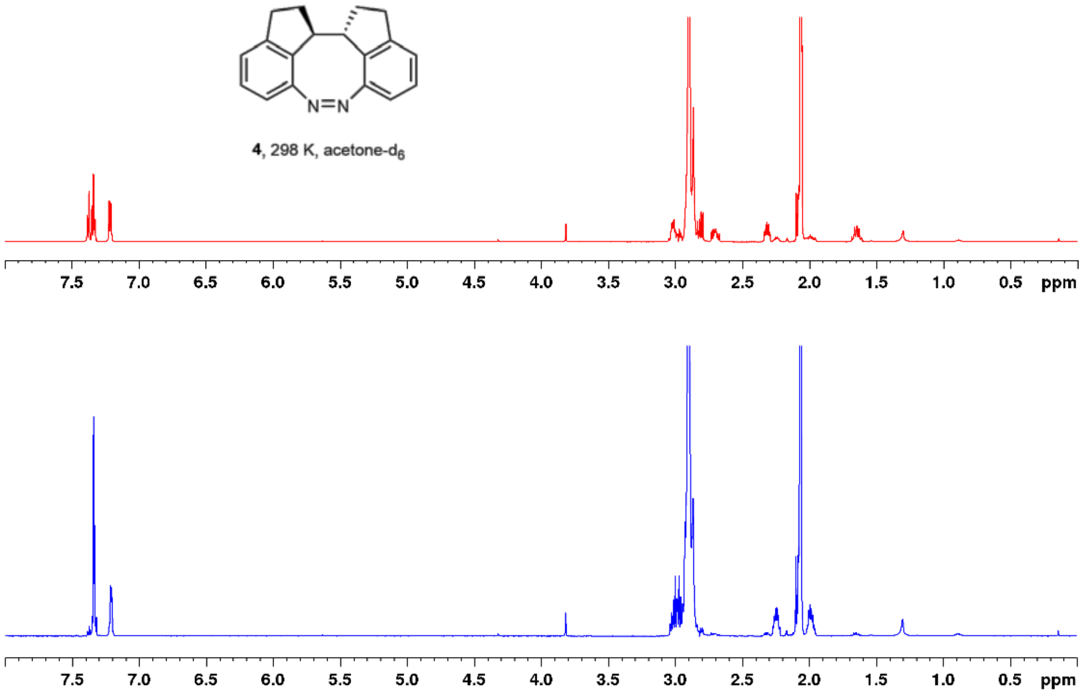

Figure S11: ^{1}H-NMR spectrum of compound DID rac **4** at PSS (385 nm) in red and PSS (530 nm) in blue both spectra measured in acetone at 298 K.

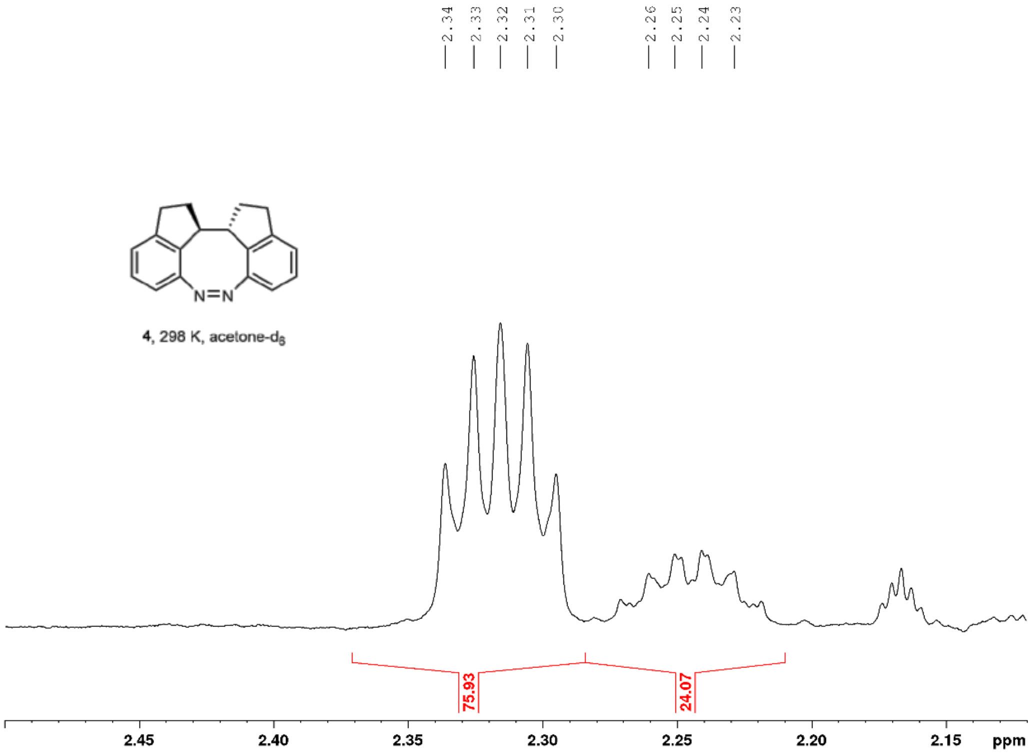

Figure S12: ^{1}H-NMR spectrum of compound DID rac **4** at PSS (385 nm) at 298 K in acetone.

6 Thermal half-lives

To determine the half-life of DID meso **3**, samples in acetone-d6 were cooled down with dry ice in acetone to 195 K and irradiated for 10 min at 385 nm. Beforehand the NMR-spectrometer was cooled down to the relevant temperature. After the NMR-tube was placed in the spectrometer spectra were recorded in appropriate intervals. The measurements were repeated at five different temperatures 233K, 238K, 240K, 243K, and 247K. The rate constants of the thermal back isomerization were determined with a first order kinetic. To determine the *cis* and *trans* species the integrals from Figure S10 were used similar to the PSS determination. The thermal-half-life at room temperature was determined with an Arrhenius plot. Figure S13 shows the thermal back isomerization at 233 K with selected spectra. For the rate constant determination, more data points were taken into calculations.

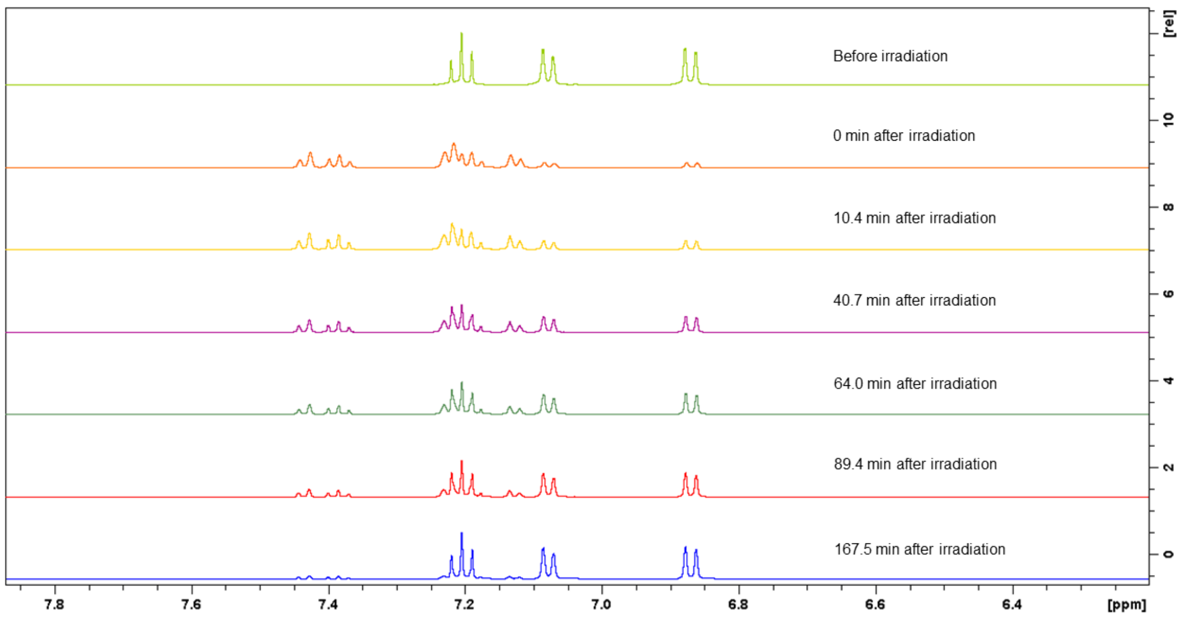

Figure S13: Thermal relaxation of DID meso **3** was recorded in the NMR-spectrometer at 233 K in acetone-d6.

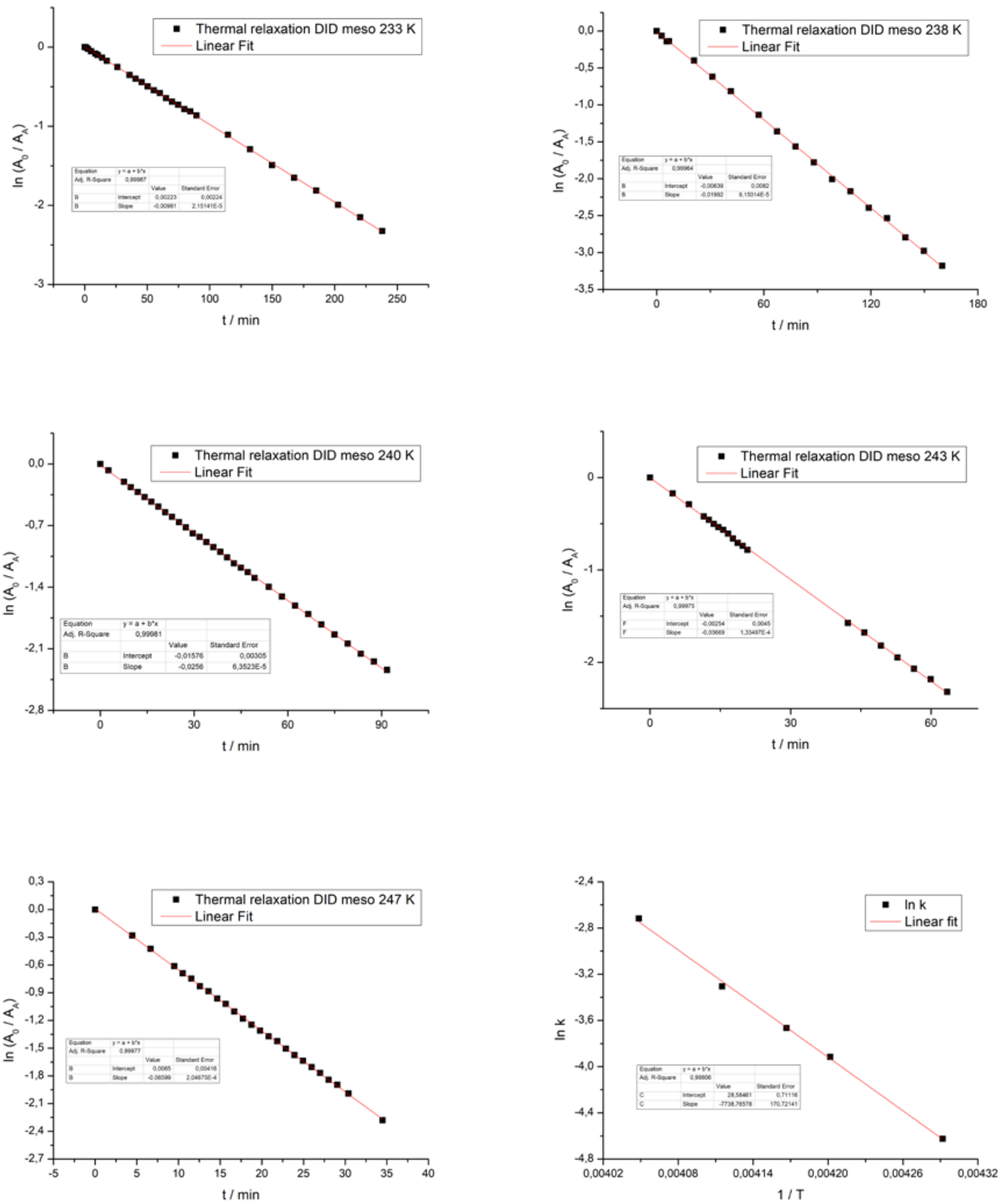

Figure S14: Thermal relaxation recorded in the NMR-spectrometer at five different temperatures. The rate constants were determined as a function of first order kinetics for five different temperatures, top left 233 K, top right 238 K, middle left 240 K, middle right 243 K and bottom left 247 K. The rate constant at room temperature was determined with an Arrhenius Plot (bottom right).

$$(A_A - A_0) = (A_0 - A_\infty) * e^{-kt}$$

Considering $A_\infty = 0$

$$\frac{A_A}{A_0} = e^{-kt}$$

$$\frac{\ln(\frac{A_0}{A_A})}{t} = k$$

$$t_{1/2} = \frac{ln2}{k}$$

Table S2: The rate constants and half-lives at given temperature of the back isomerization from *trans* to *cis* of DID meso **3**.

Temperature / K	Rate constant	Half-life $t_{1/2}$ / min
233	0.00981	70.7
238	0.01992	34.8
240	0.02560	27.1
243	0.03669	18.9
247	0.06599	10.5
300	16.2602	0.04

To determine the half-life of DID rac **4** at 300 K, a sample in acetone-d6 was irradiated for 10 min at 385 nm. After the NMR-tube was placed in the spectrometer spectra were recorded in appropriate intervals. The rate constants were determined as a function of first order kinetics. To determine the *cis* and *trans* species the integrals from Figure S15 were used similar to the PSS determination. For the rate constant determination, more data points were taken into calculations.

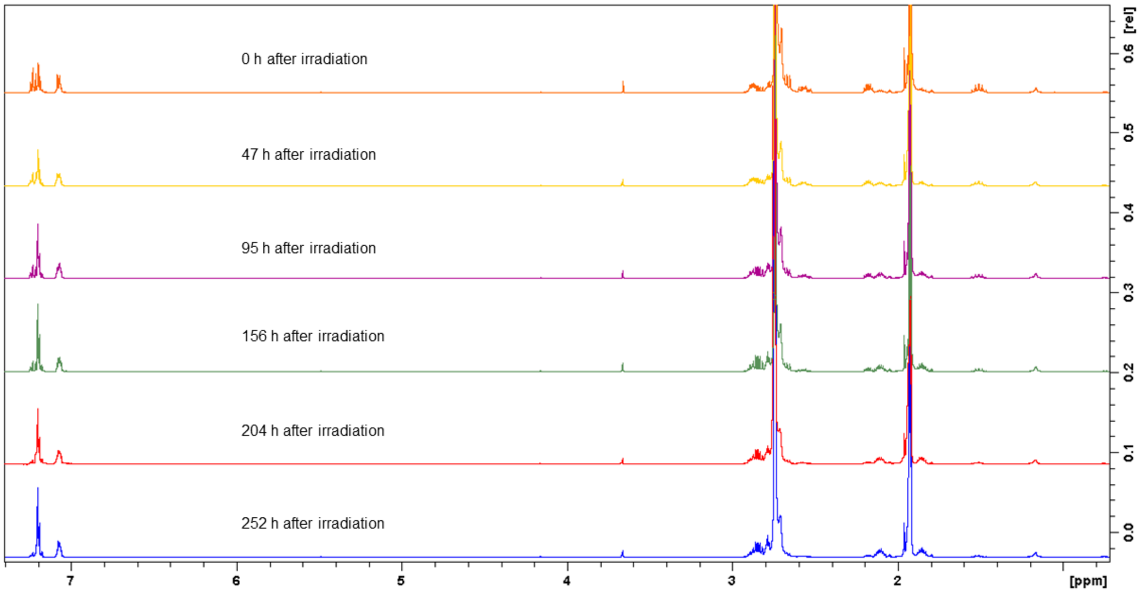

Figure S15: Thermal relaxation of DID rac **4** was recorded in the NMR-spectrometer and measured in acetone-d6.

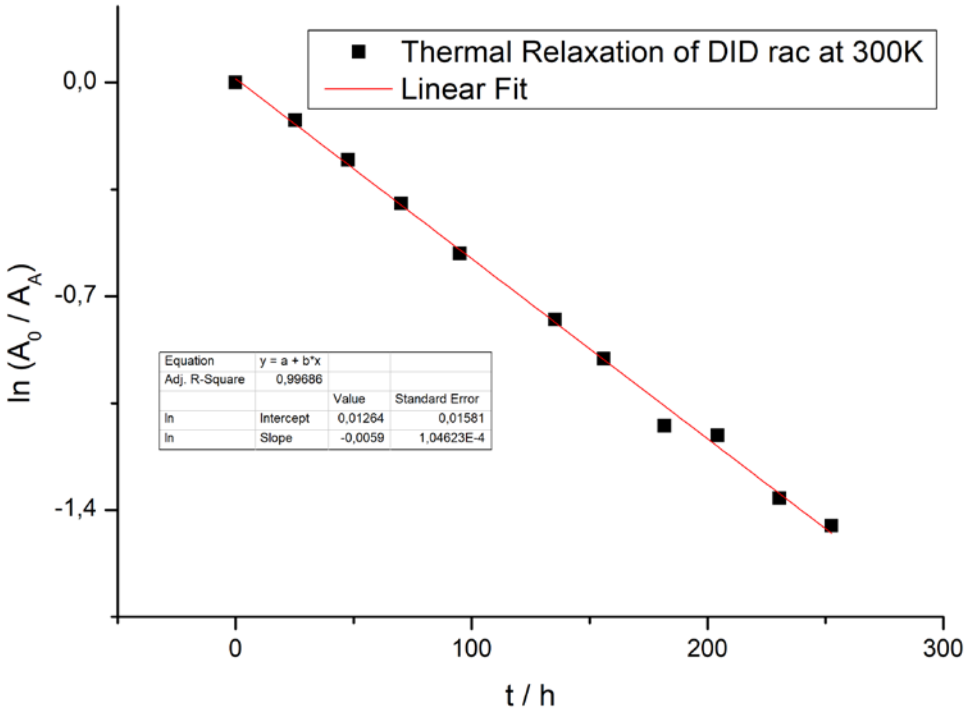

Figure S16: The rate constant of thermal relaxation of DID rac **4** at 300 K was measured with ^{1}H NMR-spectroscopy and determined as a function of first order kinetics.

$$t_{1/2} = \frac{ln2}{k} = \frac{ln2}{0.0059} = 117.5\ min$$

7 High Temperature NMR

The conformational rigidity of the diindanediazocine **3** and **4** was analyzed at higher temperatures in the NMR and compared with the parent diazocine **2**.

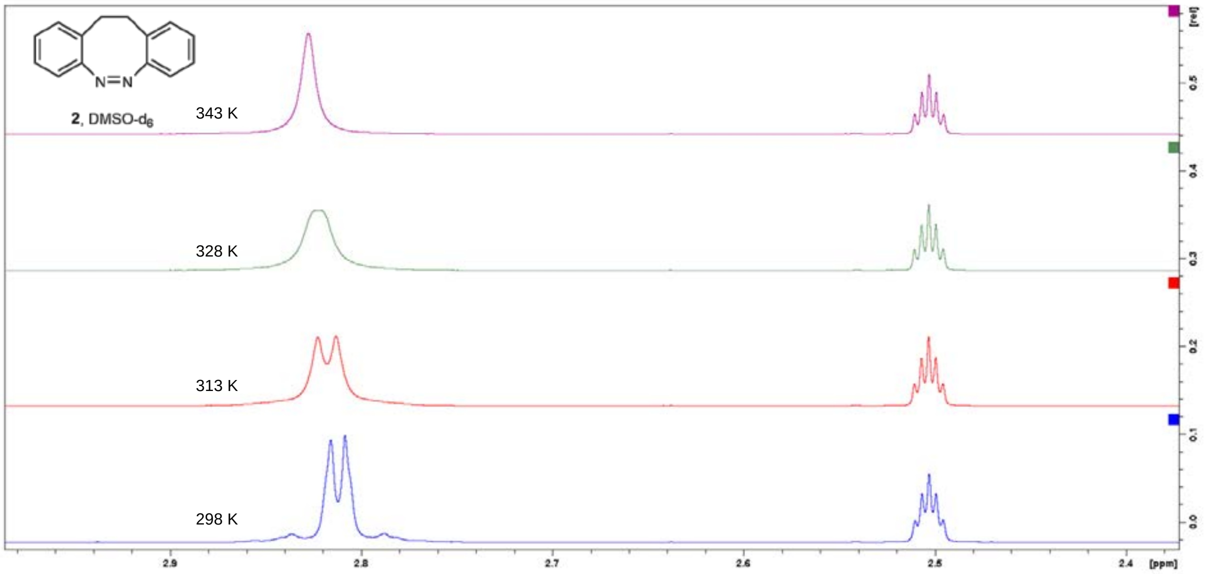

Figure S17: Upon heating from 298 K to 343 K the signal of the ethylene bridge protons of the parent diazocine **2** is increasingly broadened, confirming a boat inversion.

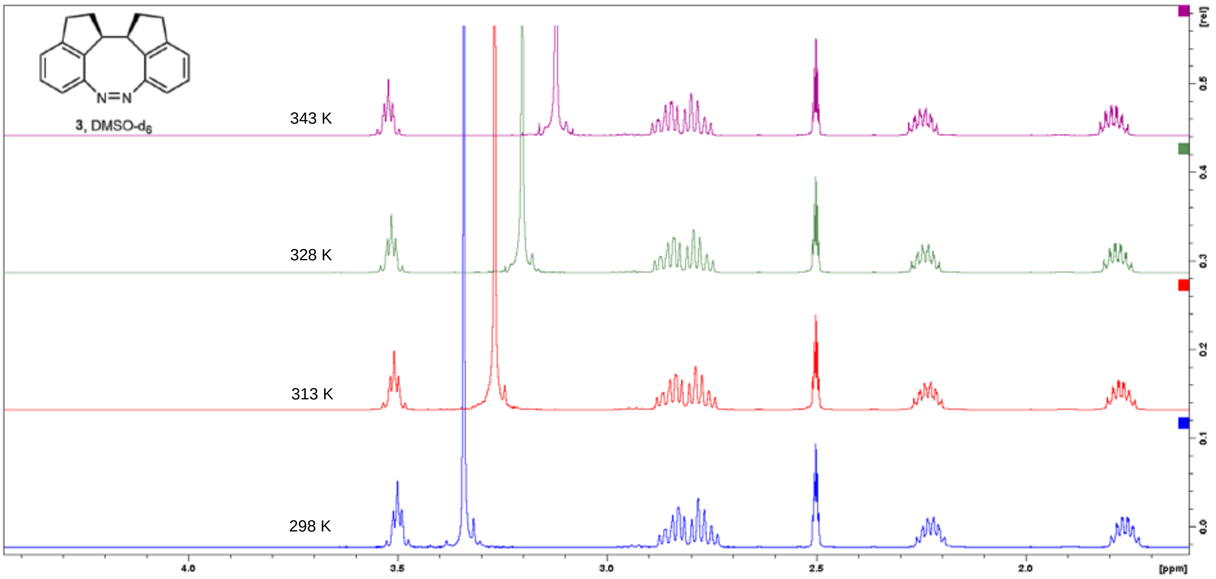

Figure S18: The ethylene bridge proton of the diindanediazocine **3** is not broadened upon heating, confirming an increased rigidity of the system.

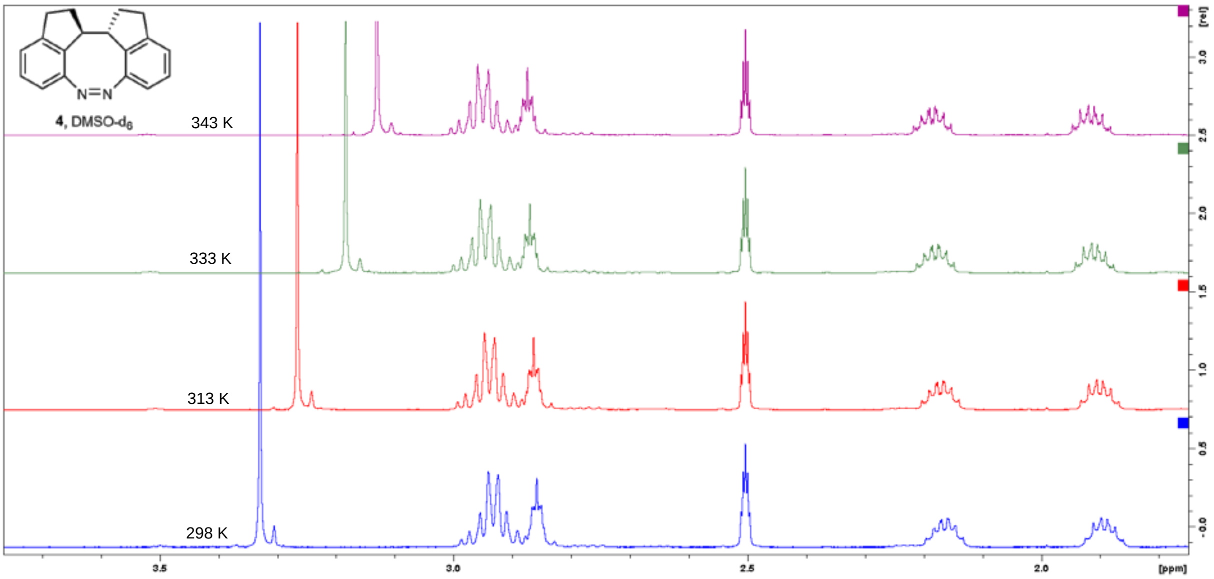

Figure S19: The ethylene bridge proton of the diindanediazocine **4** is not broadened upon heating, confirming an increased rigidity of the system.

8 Estimation of Isomerization Quantum Yields

Molar Absorption Coefficients of DID meso. The molar absorption coefficients of *cis*-**DID meso** in acetone were obtained with a calibration curve. The molar absorption coefficients of pure *trans*-**DID meso** were interpolated from a spectrum of pure *cis*-**DID meso** and a UV-Vis spectrum of the PSS reached at -15 °C with simultaneous irradiation with a 385 nm high power LED. **Figure S20a** shows the utilized spectra and **Fig. S20b** the obtained molar absorption coefficients. Increasing the LED power did not lead to observable changes in the absorption spectrum, hence it can be safely assumed that a PSS was reached. The photon flux of the LED was determined with the same irradiation geometry utilizing the ferrioxalate actinometer with on-line detection.[12]

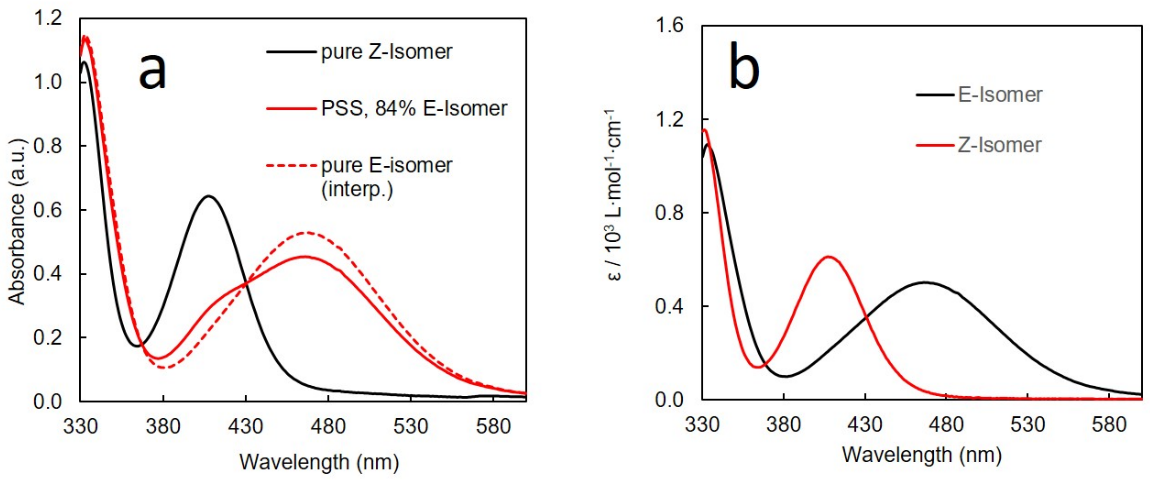

Figure S20: (a) Spectrum of pure *cis*-DID meso **3** (black), a PSS containing 84% *trans*-DID meso **3** and the interpolated spectrum of pure *trans*-**DID meso**. **(b)** Molar absorption coefficients used for quantum yield determination.

***cis* to *trans* Switching of DID meso with 385 nm.** The equilibrium between the *trans* and *cis*-isomer of compound **DID meso** is shifted to *trans* by irradiation with 385 nm (PSS with 84% *trans*-isomer). Using the method used by Rau and coworkers we obtained the quantum yields for 385 nm as the irradiation wavelength.[13-14]A degassed 2.4 mL sample containing 1.1 mM **DID meso** in acetone was irradiated *in-situ* with a 385 nm LED at different currents driving the LED (Fig. S21a). The temperature was adjusted to -4 °C and monitored during the entire experiment. After ten minutes, a spectrum of the established steady states was recorded. The

rate constant of the thermal reverse reaction was obtained by observing the reverse reaction in similar conditions (see Fig. S21b, -4°C, n = 3, k_{rev} = 0.017 s^{-1} ± 1%)

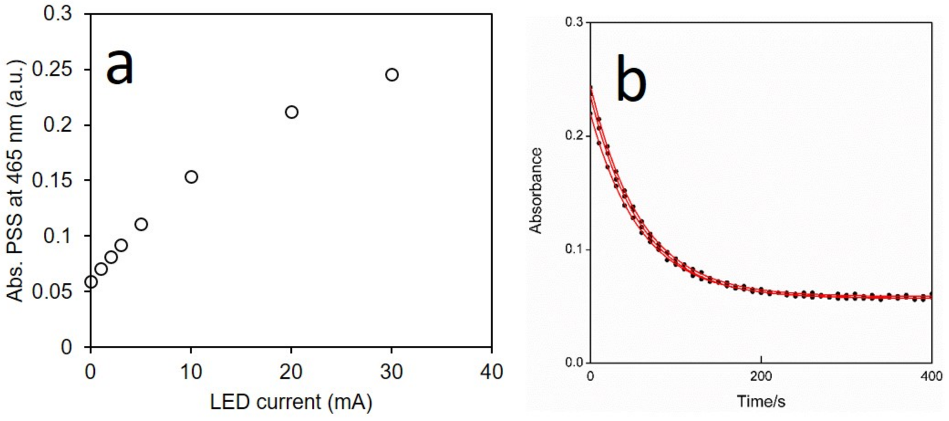

Figure S21: (a) Absorbance at 465 nm of the steady state reached with different LED currents. **(b)** Decay curves for obtaining k_{rev}.

Following Rau et al.[13] the rate of the thermal reverse reaction can be utilized to determine the switching quantum yields. In this method, the isomerization quantum yields are obtained by plotting $(A_\infty - A_o)^{-1}$ *vs.* $(q_p\, F'_\infty)^{-1}$ according to

$$\frac{1}{(A_\infty - A_o)} = \frac{Q}{(\varepsilon_{trans} - \varepsilon_{cis}) l c_0 \phi_{cis\to trans} \varepsilon'_{cis}} + \frac{k_{rev}}{(\varepsilon_{trans} - \varepsilon_{cis}) l^2 c_0 \phi_{cis\to trans} \varepsilon'_{cis}} \frac{1}{q_p F'_\infty}.$$

In this Equation quantities at the irradiation wavelength (λ', 385 nm) are primed, (*i.e.* ε'_{cis}, ε'_{trans} and F'_∞). Quantities referring to the observation wavelength ($\lambda^{obs.}$, 465 nm) are unprimed (*i.e.* A_∞, A_o, ε_{trans}, ε_{cis}). Quantities measured in a photo stationary state are subscripted with an infinity symbol (∞). Furthermore the utilized symbols have the following meaning:

F'_∞	photokinetic factor in the PSS defined as $F'_\infty = (1 - 10^{-A'_\infty})/A'_\infty$
$\phi_{cis\to trans}$, $\phi_{trans\to cis}$	quantum yields for isomerization
Q	pseudo total quantum yield, defined as $Q = \phi_{cis\to trans}\varepsilon'_{cis} + \phi_{trans\to cis}\varepsilon'_{trans}$ in L·mol^{-1}·cm^{-1}
c_0	total concentration of both isomers in mol·L^{-1}
l	optical path length (*i.e.* 1 cm) making slope and intercept a dimensionless quantity

k_{rev} rate constant for the reverse reaction (at the same temperature) in s^{-1}

q_p photon flux into the cuvette in mol·L·s^{-1}

A graph according to this Equation is shown in Fig. S22. The quantum yields are extracted from the intercept and the slope. The results are compiled in Table S3.

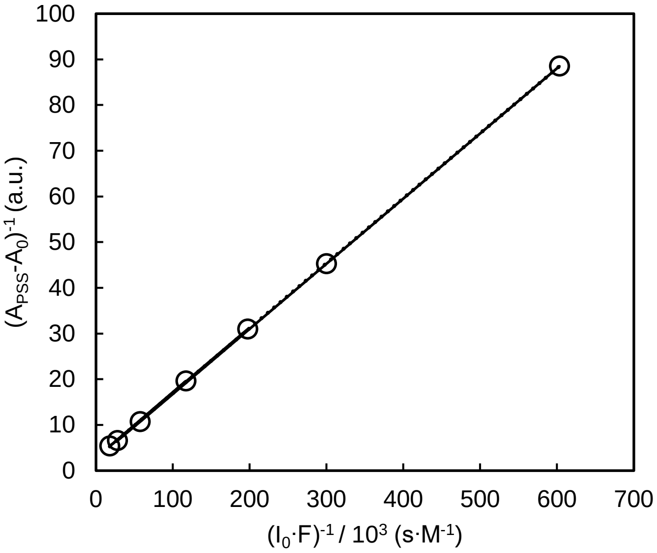

Figure S22: Plot according to the above Equation used for extracting the switching quantum yields for 385 nm irradiation.

Table S3: Data used for the plot shown in Figure S22 together with extracted isomerization quantum yields for 385 nm.

	$\lambda^{obs.}$ (465 nm)		λ' (385 nm)			
	ε_{cis}	ε_{trans}	ε'_{cis}	$\phi_{cis\rightarrow trans}$	ε'_{trans}	$\phi_{trans\rightarrow cis}$
UV (385 nm)	40	502	351	0.7	104	0.8

Switching *trans* to *cis* with 520 nm. The sample was cooled to 0 °C and pre-irradiated with a 385 nm LED to induce *cis* → *trans* conversion. After that, we accelerated the thermal *trans* → *cis* conversion with a 520 nm LED, emitting different light intensities while monitoring the absorbance at 470 nm (disappearing nπ* band of *trans*-**DID meso**). Due to the large bathochromic shift of the *trans*-form the green light is exclusively absorbed by this isomer. The overall concentration change (thermal plus photochemical reverse reaction) is hence described by

$$\frac{d[trans]}{dt} = q_p\left(1 - 10^{-A'}\right)\phi_{trans \rightarrow cis} + k_{rev}[trans].$$

The concentration change ($d[E]/dt$) is calculated with the absorbance coefficients at the irradiation wavelength as follows:

$$\frac{d[trans]}{dt} = \frac{dA}{dt}\frac{1}{(\varepsilon_{trans} - \varepsilon_{cis})}$$

The measured absorbance *vs.* time curves were fitted with a mono-exponential function and the derivative at $t = 0$ was used to determine dA/dt. The light intensity was determined with a calibrated spectrophotometer with an integrating sphere.

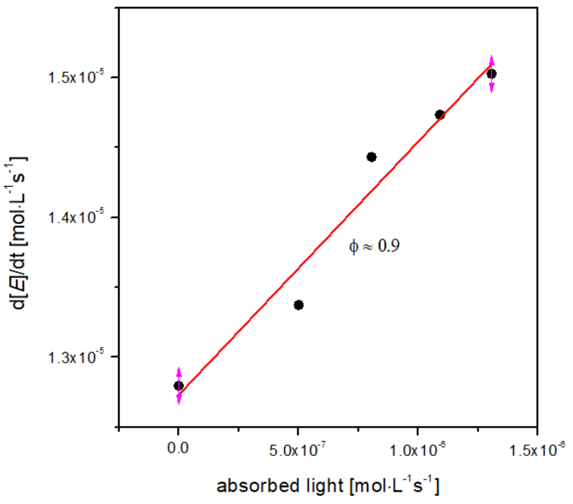

Figure S23: Absorbed light *vs.* the concentration change (*d[trans]/dt*) yields the quantum yield for 520 nm irradiation of DID meso.

Starting from a similar content of *trans*-isomer, the quantum yield for the light induced *trans* to *cis* process is obtained by plotting the absorbed photon flux ($q_p\left(1 - 10^{-A'}\right)$) *vs.* $d[trans]/dt$ (see Fig. S23). This procedure yields $\phi_{trans \rightarrow cis}$ ≈ 0.9 for DID meso. The utilized data are compiled in Table S4.

Table S4: Molar absorbance coefficients used to calculate $d[trans]/dt$ and for estimating the quantum yield.

	$\lambda^{obs.}$ (470 nm)		λ' (520 nm)	
	ε_{cis}	ε_{trans}	ε'_{trans}	$\phi_{trans\rightarrow cis}$
UV (385 nm)	29	500	252	0.9

Molar Absorption Coefficients DID rac. The molar absorption coefficients of *cis*-**DID rac** in acetone were obtained with a calibration curve. The molar absorption coefficients of pure *trans*-**DID rac** were interpolated from a spectrum of pure *cis*-**DID rac** and a UV-Vis spectrum of the PSS reached at room temperature with simultaneous irradiation with a 385 nm LED. Increasing the LED intensity did not lead to observable changes in the absorption spectrum, hence it can be safely assumed that a PSS was reached. A *trans* content of 76% was assumed. Figure S24a shows the utilized spectra and Fig. S24b the obtained molar absorption coefficients.

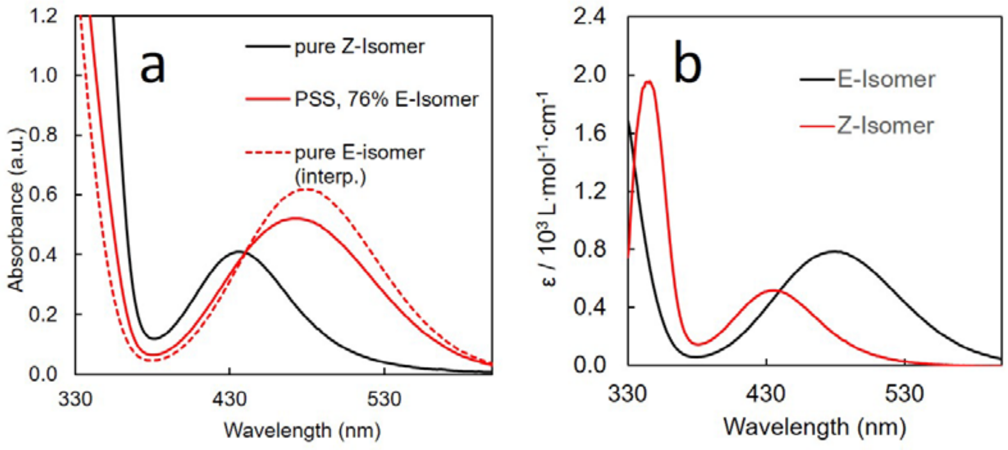

Figure S24. (a) Spectrum of pure *cis*-isomer (black) a spectrum of the PSS containing ca. 76% *trans*-isomer (red) and the interpolated spectrum of pure *trans*-isomer (red, dashed). **(b)** Molar absorption coefficients of both isomers of DID rac.

Photoisomerization with 385 nm and 520 nm. The thermal reverse reaction is slow at room temperature and negligible compared to the rate of photo-induced isomerization. Therefore, we analyzed light induced concentration (*i.e.* absorbance) changes rather than steady states reached upon prolonged light exposure. Based on the work of Gauglitz et al.[15] the temporal absorbance change in an azobenzene-type system changes according to

$$\frac{d}{dt}A = q_p F' d\, Q(A - A^{\infty}).$$

In this Equation, F' ist the photokinetic factor defined as $F'_{\infty} = (1 - 10^{-A'})/A'$ (A' is the absorbance at the irradiation wavelength) and Q is the pseudo total quantum yield, defined as $Q = \phi_{cis\rightarrow trans}\varepsilon'_{cis} + \phi_{trans\rightarrow cis}\varepsilon'_{trans}$ (in L·mol^{-1}·cm^{-1}). In the case of low absorbance (< 0.15) at the irradiation wavelength the measured absorbance *vs.* time curve is well described by an exponential function and its derivative at $t = 0$ is then: $(A - A^{\infty})k_{obs}$. Combining these two Equations offers a way for estimating the isomerization quantum yields. In a plot of k_{obs} *vs.* $q_p F'_i d$ (see Fig. S25) the pseudototal quantum yield Q is obtained.

$$k_{obs} \approx q_p F'_i d\, Q$$

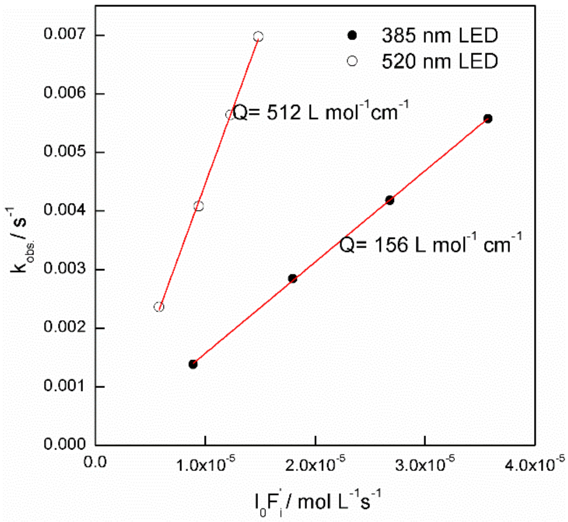

Figure S25: Plot of $q_p F'_i$ *vs.* k_{obs} for the irradiation wavelengths of 385 nm and 520 nm. The slope corresponds to the pseudototal quantum yield Q.

In combination with the *trans*-isomer content determined by NMR spectroscopy (χ_{trans}) and the extinction coefficients at the irradiation wavelength of both isomers (ε'_{cis} and ε'_{trans}) the quantum yields $\phi_{trans\rightarrow cis}$ and $\phi_{cis\rightarrow trans}$ are accessible by

$$\chi_{trans} = \frac{\varepsilon'_{cis}\phi_{cis\rightarrow trans}}{Q}.$$

The quantum yields are compiled in **Table S5**.

Table S5: Isomerization quantum yields of the parent system for 385 and 520 nm.

	χ_{trans}^{NMR}	ε'_{cis}	$\phi_{cis\rightarrow trans}$	ε'_{trans}	$\phi_{trans\rightarrow cis}$	Q
UV light (385 nm)	0.76	155	0.8	62	0.6	156
blue light (520 nm)	0.06	47	0.7	530	0.9	541

9 Single Crystal structure determinations

Single-crystal X-ray data for **4** was measured using a Rigaku SuperNova dual-source Oxford diffractometer equipped with an Eos detector using mirror-monochromated Cu-K_{α} (λ = 1.54184 Å) radiation. The data collection and reduction were performed using the program *CrysAlisPro* and Gaussian face index absorption correction method was applied.[16] The structures were solved with intrinsic phasing (SHELXT) and refined by full-matrix least squares on *F* using the *OLEX2* software, which utilises the *SHELXL*-2015 module.[17-18] Non-hydrogen atoms were assigned anisotropic displacement parameters unless stated otherwise. Hydrogen atoms were placed in idealized positions and included as riding. Isotropic displacement parameters for all H atoms were constrained to multiples of the equivalent displacement parameters of their parent atoms with U_{iso}(H) = 1.2 U_{eq} (parent atom). The X-ray single crystal data and experimental details and CCDC numbers of **3** are given below.

The data collections for **7** and **3** were performed using an Imaging Plate Diffraction System (IPDS-2) from STOE & CIE using MoK_{α}-radiation (λ = 0.71073 Å). All structure were solved with SHELXT and the structure refinements were performed using SHELXL-2018.[17-18] All non-hydrogen atoms were refined anisotropic. The C-H H atoms were positioned with idealized geometry and refined isotropic with U_{iso}(H) = 1.2 · U_{eq}(C) using a riding model.

CCDC-1979407 (**7**), CCDC-1979408 (**3**) and CCDC-1979312 (**4**) contains the supplementary crystallographic data for this paper. These data can be obtained free of charge from the Cambridge Crystallographic Data Centre via http://www.ccdc.cam.ac.uk/data_request/cif.

Table S6: Selected crystal data and details of the structure refinements for **7**, **3** and **4**.

compound	Diindan-Dinitro rac **7**	DID meso **3**	DIDrac **4**
Formula	$C_{18}H_{16}N_2O_4$	$C_{18}H_{16}N_2$	$C_{18}H_{16}N_2$
MW / g mol^{-1}	324.33	260.33	417.14
crystal system	monoclinic	Triclinic	monoclinic
space group	$P2_1/c$	P-1	$P2_1/n$
a / Å	16.0789(5)	7.7399(6)	7.9196(5)
b / Å	8.0785(3)	7.9867(6)	14.4576(6)
c / Å	12.2064(5)	11.7280(8)	11.4386(6)
α / °	90	106.063(8)	90
β / °	104.487(2)	104.013(9)	101.084(5)
γ / °	90	102.003(9)	90
V / Å^3	1535.12(10)	645.88(9)	1285.27(12)
T / K	200	200	100
Z	4	2	8
D_{calc} / g cm^{-3}	1.403	1.339	1.345
μ / mm^{-1}	0.101	0.079	0.615
θ_{max} / deg	27.085	27.000	66.734
measured refl.	21228	7309	14620
unique refl.	3346	2753	2275
R_{int}	0.0692	0.0395	0.0908
refl. [F_0>4σ(F_0)]	3024	2339	1986
parameters	217	182	181
R_1 [F_0 > 4σ(F_0)]	0.0472	0.0406	0.0442
wR_2 [all data]	0.1163	0.1104	0.1118
GOF	1.079	1.037	1.091
$\Delta\rho_{max/min}$ / e Å^{-3}	0.404 / -0.283	0.284 / -0.260	0.265 / -0.225

10 Quantum Chemical Calculations

General. All calculations were performed with Turbomole7.2,[19] the m4 grid (in Turbomole nomenclature) and the usage of resolution-of-identity (RI) with multipole accelerated RI-J (marij) to speed up the calculations. The convergence criteria for single point calculations were set to 10^{-8}. If applied, Grimmes D3 dispersion correction was combined with Becke-Johnson and zero damping factors from his latest benchmarking study for different density functionals.[20-21]All stationary points of the diazocines are characterized by frequency calculations (see coordinates).

Manuscript. All energy values in the manuscript are based on geometry optimizations using density functional theory with the standard GGA functional PBE and Ahlrich's triple zeta basis def2TZVP as it performs best in the benchmarking (see below) of azobenzene isomerization enthalpy.[22-23]

Benchmarking isomerization enthalpy of azobenzene

For an adequate description of the energy difference between *cis* and *trans* diazocines no standard quantum chemical approach is established yet. Based on the simple assumption that diazocines are electronically similar to azobenzenes, a benchmark of different standard density functional approximations (DFA) and basis sets were performed, and the results were compared with the experimentally measured isomerization enthalpy.[24-26] The experimental value was determined as 11.8 kcal mol^{-1}. From the results presented in Figure S26 the conclusion can be drawn that dispersion correction has to be included. Most of the calculated isomerization energies ($E_{calc,cis\text{-}trans}$) are more than 2 kcal mol^{-1} above the experimental values. The mean absolute deviation (MAD) for all values in this grid is 2.13 kcal mol^{-1}. By application of Grimme's dispersion correction D3 with Becke-Johnson or zero damping, the MAD goes down to 0.66 kcal mol^{-1}. Independently of the basis set PBE, BLYP and M06-L seem to perform best.

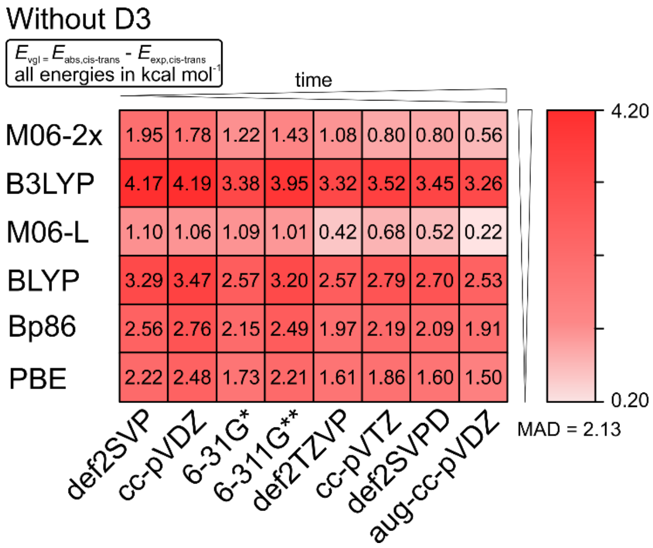

Figure S26: Deviation of DFT values from experimental result (11.8 kcal mol^{-1}) for isomerisation enthalpy difference of azobenzene. Different standard DFAs and basis sets were applied without any additive dispersion correction. Most expensive is top right und cheapest down left.

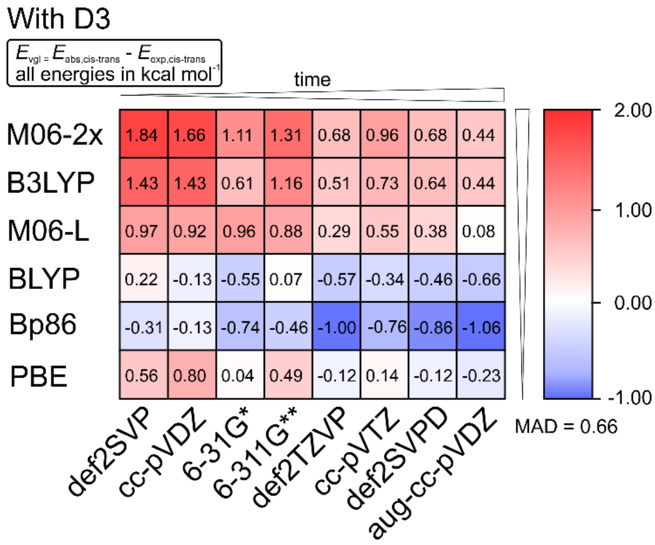

Figure S27: Deviation of DFT values from experimental result (11.8 kcal mol^{-1}) for isomerisation enthalpy difference of azobenzene. Different standard DFAs and basis sets were applied with Grimme’s D3 dispersion correction. Most expensive is top right und cheapest down left.

Overlay of DFT and crystal structures

To estimate the quality of the geometry optimizations, overlays of the structures with the here presented crystal structures of DID rac **3** and meso **4** (each in *trans*) were performed. The most promising combinations PBE(D3BJ)/def2TZVP, BLYP(D3BJ)/6-311G** and M06-L(D3zero)/def2TZVP were included and the resulting deviations are shown in Table S7. The best result is provided by the combination of PBE(D3BJ) with the triple zeta basis def2TZVP.

Table S7: Deviation of DFT geometries in a crystal structure overlay for *trans*-DID rac **3** and meso **4**. Listed are the three most promising combinations of the energy benchmark. All values in Angstrom.

	rac **3**		meso **4**	
Niveau\DID	RMSD	max. D	RMSD	max. D
M06-L(D3zero)/def2TZVP	0.1118	0.1949	0.0663	0.1255
BLYP(D3BJ)/6-311G**	0.1212	0.2035	0.0530	0.1043
PBE(D3BJ)/def2TZVP	0.0960	0.1680	0.0501	0.1042

Coordinates of PBE(D3BJ)/def2TZVP structures

DAC 2 - *Z*

$E_{PBE(D3BJ)/def2TZVP}$ = -649.6114825558

NImag = 0

C	0.7949929	2.0794689	0.0622244
H	0.7568154	2.4736812	1.0877836
H	1.3547910	2.8128565	-0.5359163
C	-3.0158648	-0.9309994	-1.1083668
C	-2.2254172	0.2037893	-1.2695423
C	-1.4067860	0.7059207	-0.2448867
C	-1.4097993	0.0042364	0.9769437
C	-2.2384556	-1.1128917	1.1611509
C	-3.0216976	-1.5963024	0.1191083
C	1.5247829	0.7655207	0.0640158
N	-0.6978280	0.3951974	2.1698884
N	0.5432780	0.2702552	2.2567966
C	1.3275151	-0.1355216	1.1267775
C	2.4071216	0.3919336	-0.9553971
C	3.0616765	-0.8400049	-0.9319231
C	2.8619977	-1.7141913	0.1401101
C	2.0158248	-1.3513497	1.1856301
H	-3.6297517	-1.2897713	-1.9354923
H	-2.2291006	0.7283723	-2.2281135
H	-2.2427429	-1.5975480	2.1387181
H	2.5791501	1.0846706	-1.7829248
H	3.7396809	-1.1121863	-1.7419107
H	1.8698108	-2.0051539	2.0466967
C	-0.6403831	1.9822806	-0.5086144
H	-1.2238100	2.8397212	-0.1308350
H	-0.5946946	2.1055240	-1.6008961
H	-3.6394300	-2.4827635	0.2690661
H	3.3783235	-2.6747448	0.1699089

DAC 2 – *E*-twist

$E_{PBE(D3BJ)/def2TZVP}$ = -649.5990525187

NImag = 0

C	0.5310547	-0.5736432	1.3396743
H	0.0312698	-1.5263510	1.1067287
H	0.8955306	-0.6765015	2.3729754
C	-4.1992759	0.4959548	0.2121170
C	-3.0456204	0.6837843	0.9779260
C	-1.7732240	0.4105909	0.4655640
C	-1.7089954	-0.0785443	-0.8634078
C	-2.8562531	-0.3239676	-1.6193149
C	-4.1054463	-0.0096223	-1.0871764
C	1.7732295	-0.4109121	0.4654966
N	-0.4089830	-0.4807581	-1.2436148
N	0.4088992	0.4803597	-1.2436541
C	1.7089289	0.0781541	-0.8634967
C	3.0456741	-0.6837600	0.9779215
C	4.1993280	-0.4954770	0.2122203
C	4.1054179	0.0100081	-1.0871033
C	2.8561507	0.3238391	-1.6193728
H	-5.1761365	0.7256680	0.6399750
H	-3.1331687	1.0474956	2.0048191
H	-2.7555078	-0.7350296	-2.6246718
H	3.1332554	-1.0476448	2.0047503
H	5.1762448	-0.7247223	0.6402009
H	2.7553243	0.7346303	-2.6248326
C	-0.5310041	0.5733694	1.3396697
H	-0.0312295	1.5260640	1.1066472
H	-0.8954276	0.6762846	2.3729834
H	5.0061047	0.1698794	-1.6814346
H	-5.0061398	-0.1691487	-1.6815900

DAC 2 – *E*-chair

$E_{PBE(D3BJ)/def2TZVP}$ = -649.5940446212

NImag = 0

C	4.0136998	-1.1841560	-0.0321606
C	2.7312744	-1.6907099	0.2012646
C	1.6797821	-0.7932776	0.3428663
C	4.2130211	0.1927249	-0.1428013
C	3.1367758	1.0777944	-0.0093031
C	1.8487251	0.6081587	0.2564593
C	0.6527600	1.5260875	0.4492538
C	-0.5769276	1.3456485	-0.5079523
C	-1.7698544	0.4522332	-0.1584088
C	-1.7043508	-0.9669897	-0.1753720
C	-3.0430649	1.0159883	-0.0122798
C	-4.1877853	0.2233929	0.1057563
C	-4.0913065	-1.1688134	0.0378870
C	-2.8429833	-1.7685359	-0.1250885
N	0.3121156	-1.0376552	0.6061365
N	-0.3836494	-1.4673643	-0.3497827
H	2.5461082	-2.7627492	0.2756846
H	5.2137080	0.5841795	-0.3311593
H	3.3008319	2.1541866	-0.1027947
H	0.2989701	1.4372345	1.4886004
H	1.0140939	2.5583863	0.3220626
H	-0.1953703	1.0212245	-1.4905952
H	-0.9968005	2.3502653	-0.6687241
H	-3.1393295	2.1045946	-0.0070477
H	-5.1629574	0.6973139	0.2270901
H	-2.7363342	-2.8529753	-0.1757630
H	-4.9871698	-1.7866273	0.1137630
H	4.8560178	-1.8695595	-0.1375915

DID meso 3 - *Z*

$E_{PBE(D3BJ)/def2TZVP}$ = -804.3213674079

NImag = 0

N	1.5560091	-1.9044388	-1.4835633
N	1.2963049	-2.4977620	-0.4145962
C	0.8560555	-1.8264133	0.7693344
C	-0.4172764	-1.2516101	0.8112855
C	-1.3511962	-1.0267913	-0.3449662
C	0.5370028	0.4270462	-1.4394586
C	1.6056120	-0.4702364	-1.5736300
C	1.5740608	-1.9827885	1.9648818
C	1.0327002	-1.5208359	3.1643302
C	-0.2595438	-0.9790496	3.2134071
C	-0.9901287	-0.8716123	2.0312290
C	2.8772793	0.0194400	-1.9258622
C	3.1057146	1.3841474	-2.0649896
C	2.0536266	2.2919581	-1.9030754
C	0.7866544	1.8047020	-1.6026435
C	-2.7245952	-0.8933448	0.3689112
H	2.5496941	-2.4702202	1.9422716
H	1.6116785	-1.6215088	4.0838401
H	-0.6917397	-0.6766455	4.1692436
C	-2.4161622	-0.4181145	1.8126950
H	3.6845164	-0.7010360	-2.0650066
H	4.1087616	1.7406914	-2.3040565
H	2.2240047	3.3648884	-2.0112072
C	-0.4847625	2.5879567	-1.3930662
H	-0.3250709	3.5343357	-0.8570984
H	-0.9424430	2.8388287	-2.3671478
H	-3.1940424	-1.8866450	0.4122361
H	-3.1059504	-0.8549366	2.5496717
H	-2.5044147	0.6762632	1.9115267
C	-1.3632923	1.5879299	-0.6247735
H	-1.1003145	1.6314629	0.4435294
H	-2.4381710	1.7868517	-0.7265448
H	-1.3475656	-1.8979561	-1.0187254
H	-3.4193331	-0.2292699	-0.1621566
C	-0.9480696	0.2085824	-1.1882667
H	-1.4356034	0.0961411	-2.1775685

DID meso 3 – *E*-twist

$E_{PBE(D3BJ)/def2TZVP}$ = -804.2919405528

NImag = 0

N	-2.0147465	-0.3888807	0.1261070
N	-1.5386240	-1.5270690	-0.1511394
C	-0.8973716	-2.0185315	1.0317955
C	0.3155892	-1.3492036	1.2277229
C	1.1306729	-0.4632473	0.2836445
C	0.6490198	0.0682109	-1.1072507
C	-0.6739593	0.7780691	-1.4186203
C	-1.9743796	0.5178928	-0.9519637
C	-1.3660846	-2.9321653	1.9734739
C	-0.6462668	-3.1010890	3.1624700
C	0.4997151	-2.3397931	3.4294810
C	0.9692856	-1.4536776	2.4597904
C	-3.0560610	1.2961585	-1.3793589
C	-2.8517961	2.2793577	-2.3471270
C	-1.5725115	2.5151593	-2.8663837
C	-0.4929689	1.7677861	-2.3941312
C	1.7819954	0.4899996	1.3142316
H	0.6432388	-0.8290622	-1.7524552
H	-2.3133916	-3.4473740	1.8117359
H	-1.0215135	-3.7934466	3.9178424
H	1.0005731	-2.4262791	4.3958019
C	2.1033366	-0.4542540	2.5002773
H	-4.0474455	1.1092228	-0.9651636
H	-3.6982654	2.8709278	-2.6994993
H	-1.4220409	3.2834053	-3.6275083
C	0.9470181	1.7999403	-2.8415574
C	1.6767432	1.0700618	-1.7011994
H	1.3271695	2.8166329	-3.0169241
H	1.0492774	1.2493230	-3.7935395
H	1.9398245	1.8063520	-0.9276352
H	2.6052456	0.5769620	-2.0214614
H	1.0567395	1.2583891	1.6272240
H	2.6802466	0.9930929	0.9337517
H	3.0728542	-0.9561455	2.3270334
H	2.1767528	0.0664199	3.4654442
H	1.9621287	-1.1331454	-0.0249096

DID meso 3 – TS *E* → *Z*

$E_{PBE(D3BJ)/def2TZVP}$ = -804.2683817917

NImag = -515.98 cm^{-1}

C	-0.5454497	-0.7696501	-0.7948098
C	0.4574662	3.8439016	0.1796317
C	-0.2110549	2.7843876	-0.4522110
C	-0.5353593	1.6341551	0.2463205
C	-0.2267096	1.5044940	1.6289308
C	0.4269644	2.5760877	2.2817800
C	0.7624903	3.7117754	1.5395397
C	-0.1247740	-1.7214908	0.3339924
N	-0.4949730	0.2904823	2.0985452
N	-1.1397451	-0.7306117	2.3539147
C	-0.5067177	-1.8550865	1.6811043
C	0.7409095	-2.6859818	-0.2066884
C	1.1283679	-3.8030040	0.5343537
C	0.6382149	-3.9745240	1.8344298
C	-0.1794246	-3.0003232	2.4101742
H	0.7371136	4.7444435	-0.3677761
H	0.6896392	2.4919497	3.3353287
H	1.2766548	4.5266369	2.0532759
H	1.8127409	-4.5382633	0.1057742
H	0.9312796	-4.8486448	2.4176009
H	-0.5081516	-3.0764153	3.4478758
C	-1.2484031	0.5935577	-0.5656689
H	-2.2062784	0.3430512	-0.0758517
C	-0.6197833	2.6162310	-1.8994386
H	0.2736142	2.4562900	-2.5270968
H	-1.1294587	3.5050748	-2.2999010
C	-1.5448783	1.3710408	-1.8869598
H	-1.4173999	0.7419999	-2.7786608
H	-2.5937171	1.6986360	-1.8764422
C	0.7138731	-0.8090410	-1.6835885
H	1.4910524	-0.1614256	-1.2470578
H	0.5210450	-0.4731504	-2.7109857
C	1.1437866	-2.2899300	-1.6086789
H	2.2134871	-2.4518786	-1.8034286
H	0.5854694	-2.8855499	-2.3537930
H	-1.3118906	-1.3592244	-1.3435349

DID rac 4 - *Z*

$E_{PBE(D3BJ)/def2TZVP}$ = -804.3157176209

NImag = 0

N	-2.6685891	0.4754977	0.4955684
N	-2.3970446	-0.2904108	1.4413810
C	-1.2828324	-1.1627717	1.5511554
C	0.0325492	-0.6954857	1.5842747
C	0.5669145	0.6492930	1.1557423
C	0.6420406	0.6227008	-0.3805666
C	-0.6651540	0.7833616	-1.1386792
C	-2.0201566	0.6174958	-0.7840617
C	-1.5406051	-2.4974799	1.9184858
C	-0.4914476	-3.3522250	2.2419546
C	0.8299908	-2.8827961	2.2803351
C	1.0792217	-1.5503236	1.9641850
C	-2.9995430	0.8385911	-1.7804876
C	-2.6664072	1.1673328	-3.0862601
C	-1.3273595	1.4032149	-3.4185960
C	-0.3556917	1.2403771	-2.4393425
C	1.9540129	0.6711805	1.8439583
H	1.0274072	-0.3804424	-0.6564457
H	-2.5755011	-2.8412605	1.9494296
H	-0.7063314	-4.3907162	2.4990245
H	1.6437686	-3.5502347	2.5704516
C	2.3941223	-0.8073275	1.9164663
H	-4.0429925	0.7656894	-1.4704984
H	-3.4531813	1.3054431	-3.8291754
H	-1.0537766	1.7456313	-4.4187710
C	1.1221765	1.5266101	-2.5574911
C	1.5561567	1.6426646	-1.0861209
H	1.3361115	2.4227299	-3.1568650
H	1.6428533	0.6801569	-3.0413025
H	1.3445664	2.6562114	-0.7069738
H	2.6236216	1.4339510	-0.9357971
H	1.8286043	1.0612522	2.8649087
H	2.6797285	1.3162507	1.3325062
H	2.9664869	-1.1002593	1.0188198
H	3.0372245	-1.0194270	2.7825838
H	-0.0609446	1.4955245	1.4762028

DID rac 4 – *E*-twist

$E_{PBE(D3BJ)/def2TZVP}$ = -804.3101643461

NImag = 0

N	-0.6084497	-0.1695970	-2.0635565
N	0.6082652	0.1694288	-2.0636190
C	1.4496942	-0.8939184	-1.6634436
C	1.2256421	-1.2792400	-0.3304543
C	0.1843845	-0.7571228	0.6686609
C	-0.1844654	0.7569990	0.6686790
C	-1.2256921	1.2791686	-0.3304422
C	-1.4497902	0.8938346	-1.6634229
C	2.5387654	-1.4006825	-2.3786218
C	3.3598722	-2.3580197	-1.7805277
C	3.1339238	-2.7763314	-0.4624167
C	2.0776433	-2.2199580	0.2595332
C	-2.5388277	1.4006812	-2.3785956
C	-3.3598104	2.3581367	-1.7805219
C	-3.1337938	2.7764845	-0.4624349
C	-2.0775725	2.2200099	0.2595240
C	0.8341472	-1.1999299	2.0103850
H	0.7539134	1.3260048	0.5284345
H	2.7197982	-1.0609496	-3.3989515
H	4.1949774	-2.7758330	-2.3451454
H	3.7956170	-3.5113903	0.0001375
C	1.6555524	-2.4620594	1.6868216
H	-2.7199382	1.0609052	-3.3988977
H	-4.1948798	2.7760194	-2.3451411
H	-3.7953928	3.5116424	0.0000965
C	-1.6553692	2.4621401	1.6867725
C	-0.8343015	1.1997995	2.0103715
H	-2.5028339	2.6129590	2.3705265
H	-1.0250478	3.3669938	1.7478056
H	-1.5226565	0.4054531	2.3428413
H	-0.0970837	1.3584634	2.8078999
H	0.0968558	-1.3588288	2.8077989
H	1.5222755	-0.4054595	2.3430277
H	2.5030853	-2.6125966	2.3705534
H	1.0254818	-3.3670808	1.7479583
H	-0.7539890	-1.3261267	0.5283648

DID rac 4 – TS *E* → *Z*

$E_{PBE(D3BJ)/def2TZVP}$ = -804.2804531391

NImag = -455.79 cm^{-1}

C	0.3400115	-0.5613275	-0.6225438
H	1.3440437	-0.1241108	-0.4543202
C	-0.0499320	4.1788203	0.3711512
C	-0.4337874	3.0311135	-0.3283192
C	-0.3628301	1.7752887	0.2673433
C	-0.0496000	1.6515025	1.6481639
C	0.3791354	2.8115825	2.3543019
C	0.3961030	4.0361727	1.6971355
C	0.1744631	-1.7158964	0.3792345
N	-0.2168648	0.4388989	2.1458068
N	-0.6990855	-0.6455341	2.4865665
C	-0.2446395	-1.7775354	1.7248973
C	0.5404235	-2.9297428	-0.2315524
C	0.5005563	-4.1478485	0.4484270
C	0.0527682	-4.1875124	1.7714247
C	-0.3349064	-3.0062393	2.3948354
H	-0.0615472	5.1596408	-0.1058843
H	0.6928469	2.7212964	3.3939859
H	0.7385071	4.9171809	2.2437131
H	0.7990022	-5.0663532	-0.0615703
H	-0.0039074	-5.1360071	2.3072996
H	-0.7111116	-2.9947963	3.4191632
C	-0.6564552	0.6396212	-0.6805514
H	-1.6758290	0.2361460	-0.5206959
C	-0.9051308	2.8712933	-1.7519566
H	-0.4105071	3.5557669	-2.4566402
H	-1.9889624	3.0729708	-1.8121157
C	-0.5990652	1.3883069	-2.0486225
H	0.4290910	1.3226834	-2.4359778
H	-1.2654491	0.9512627	-2.8037624
C	0.3620621	-1.3352251	-1.9698014
H	0.9003225	-0.7962085	-2.7583717
H	-0.6783593	-1.4725732	-2.3080046
C	0.9742365	-2.7027215	-1.6545183
H	2.0769588	-2.6614985	-1.7090377
H	0.6474382	-3.4984177	-2.3392034

11 Summary of Photophysical Properties

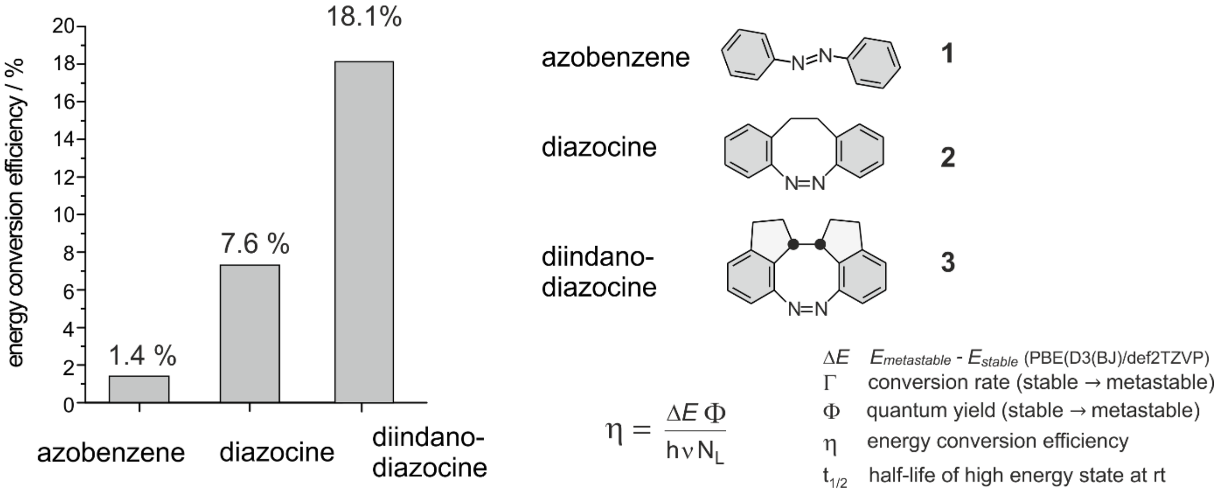

		λ_{max} [nm]	$h\nu N_L$ [kcal/mol]	Γ	Φ	Δ*E*	η	$t_{1/2}$
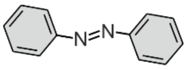	*E*→*Z*	317	90.2	92%	11%	11.7	1.4 %	5.7 h
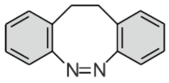	*Z*→*E*	385	74.3	92%	72%	7.6	7.3 %	27.6 h
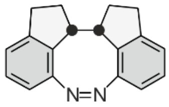	*Z*→*E*	400	71.5	84%	70%	18.5	18.1 %	3 s

Figure S28: Photophysical properties of **3** and **4**, and light-to-chemical-energy conversion efficiency.

12 Literature

1. Santos, C. S., Miller, A. C., Pace, T. C. S., Morimitsu, K. & Bohne, C. Photochromism of a spiropyran and a diarylethene in bile salt aggregates in aqueous solution. *Langmuir : the ACS journal of surfaces and colloids* **30**, 11319–11328 (2014).

2. Irie, M., Sakemura, K., Okinaka, M. & Uchida, K. Photochromism of Dithienylethenes with Electron-Donating Substituents. *J. Org. Chem.* **60**, 8305–8309 (1995).

3. Deblauwe, V. & Smets, G. *Makromol. Chem.* **189**, 2503–2512 (1988).

4. Maerz, B. et al. Making fast photoswitches faster--using Hammett analysis to understand the limit of donor-acceptor approaches for faster hemithioindigo photoswitches. *Chemistry* (Weinheim an der Bergstrasse, Germany) **20**, 13984–13992 (2014).

5. Petermayer, C., Thumser, S., Kink, F., Mayer, P. & Dube, H. Hemiindigo. Highly Bistable Photoswitching at the Biooptical Window. *J. Am. Chem. Soc.* **139**, 15060–15067 (2017).

6. Conyard, J., Cnossen, A., Browne, W. R., Feringa, B. L. & Meech, S. R. Chemically optimizing operational efficiency of molecular rotary motors. *J. Am. Chem. Soc.* **136**, 9692–9700 (2014).

7. Malkin, S. & Fischer, E. Temperature Dependence of Photoisomerization. III. 1 Direct and Sensitized Photoisomerization of Stilbenes. *J. Phys. Chem.* **68**, 1153–1163 (1964).

8. Rodier, J. M. & Myers, A. B. cis-Stilbene photochemistry. Solvent dependence of the initial dynamics and quantum yields. *J. Am. Chem. Soc.* **115**, 10791–10795 (1993).

9. Bortolus, P. & Monti, S. *Cis-trans* photoisomerization of azobenzene. Solvent and triplet donors effects. *J. Phys. Chem.* **83**, 648–652 (1979).

10. Jevric, M. et al. Norbornadiene-Based Photoswitches with Exceptional Combination of Solar Spectrum Match and Long-Term Energy Storage. *Chemistry* (Weinheim an der Bergstrasse, Germany) **24**, 12767–12772 (2018).

11. Siewertsen, R. et al. Highly efficient reversible Z-E photoisomerization of a bridged azobenzene with visible light through resolved S(1)(n pi*) absorption bands. *J. Am. Chem. Soc.* **131**, 15594–15595 (2009).

12. Lehóczki, T., Józsa, É. & Ősz, K. Ferrioxalate actinometry with online spectrophotometric detection. *Journal of Photochemistry and Photobiology A: Chemistry* **251**, 63–68 (2013).

13. Rau, H., Greiner, G., Gauglitz, G. & Meier, H. Photochemical quantum yields in the A (+h.nu.) .dblarw. B (+h.nu.,.DELTA.) system when only the spectrum of A is known. *J. Phys. Chem.* **94**, 6523–6524 (1990).

14. Siewertsen, R., Schönborn, J. B., Hartke, B., Renth, F. & Temps, F. Superior Z→E and E→Z photoswitching dynamics of dihydrodibenzodiazocine, a bridged azobenzene, by S1(nπ*) excitation at λ = 387 and 490 nm. *PCCP* **13**, 1054–1063 (2011).

15. Gauglitz, G. *J. Photochem.* **5**, 41–47 (1976).

16. Rigaku Oxford Diffraction, 2018, *CrysAlisPro* Software system, version 38.46, Rigaku Corporation, Oxford, UK.

17. Sheldrick, G. M. SHELXT - integrated space-group and crystal-structure determination. *Acta crystallographica*. **71**, 3–8 (2015).

18. Dolomanov, O. V., Bourhis, L. J., Gildea, R. J., Howard, J. A. K. & Puschmann, H. OLEX2. A complete structure solution, refinement and analysis program. *J. Appl. Crystallogr.* **42**, 339–341 (2009).

19.TURBOMOLE V7.2 2017, a development of University of Karlsruhe and Forschungszentrum Karlsruhe GmbH, 1989-2007, TURBOMOLE GmbH, since 2007; available from http://www.turbomole.com.

20. Grimme, S., Antony, J., Ehrlich, S. & Krieg, H. A consistent and accurate ab initio parametrization of density functional dispersion correction (DFT-D) for the 94 elements H-Pu. *The Journal of chemical physics* **132**, 154104 (2010).

21. Goerigk, L. et al. A look at the density functional theory zoo with the advanced GMTKN55 database for general main group thermochemistry, kinetics and noncovalent interactions. *PCCP* **19**, 32184–32215 (2017).

22. Perdew, Burke & Ernzerhof. Generalized Gradient Approximation Made Simple. *Phys. Rev. Lett.* **77**, 3865–3868 (1996).

23. Weigend, F., Häser, M., Patzelt, H. & Ahlrichs, R. RI-MP2. Optimized auxiliary basis sets and demonstration of efficiency. *Chem. Phys. Lett.* **294**, 143–152 (1998).

24. Haberfield, P., Block, P. M. & Lux, M. S. Enthalpies of solvent transfer of the transition states in the cis-trans isomerization of azo compounds. Rotation vs. the nitrogen inversion mechanism. *J. Am. Chem. Soc.* **97**, 5804–5806 (1975).

25. Adamson, A. W., Vogler, A., Kunkely, H. & Wachter, R. Photocalorimetry. Enthalpies of photolysis of trans-azobenzene, ferrioxalate and cobaltioxalate ions, chromium hexacarbonyl, and dirhenium decarbonyl. *J. Am. Chem. Soc.* **100**, 1298–1300 (1978).

26. Dias, A. R. et al. Enthalpies of formation of cis-azobenzene and trans-azobenzene. The *Journal of Chemical Thermodynamics* **24**, 439–447 (1992).
